# Thermal decomposition of [Co(*en*)_3_][Fe(CN)_6_]∙ 2H_2_O: Topotactic dehydration process, valence and spin exchange mechanism elucidation

**DOI:** 10.1186/1752-153X-7-28

**Published:** 2013-02-08

**Authors:** Zdeněk Trávníček, Radek Zbořil, Miroslava Matiková-Maľarová, Bohuslav Drahoš, Juraj Černák

**Affiliations:** 1 Regional Centre of Advanced Technologies and Materials & Department of Inorganic Chemistry, Palacký University, Tř, 17. listopadu 12, Olomouc, CZ-77146, Czech Republic; 2 Regional Centre of Advanced Technologies and Materials & Department of Physical Chemistry, Palacký University, Tř, 17. listopadu 12, Olomouc, CZ-77146, Czech Republic; 3 Department of Inorganic Chemistry, Institute of Chemistry, Faculty of Sciences, P.J. Šafárik University in Košice, Moyzesova 11, Košice, SK-041 54, Slovakia

**Keywords:** Hexacyanidoferrate, Crystal structure, Thermal behavior, Mössbauer spectroscopy, Topotactic dehydration, Nanocomposite particles, CoFe_2_O_4_

## Abstract

**Background:**

The Prussian blue analogues represent well-known and extensively studied group of coordination species which has many remarkable applications due to their ion-exchange, electron transfer or magnetic properties. Among them, Co-Fe Prussian blue analogues have been extensively studied due to the photoinduced magnetization. Surprisingly, their suitability as precursors for solid-state synthesis of magnetic nanoparticles is almost unexplored.

In this paper, the mechanism of thermal decomposition of [Co(*en*)_3_][Fe(CN)_6_] ∙∙ 2H_2_O (1a) is elucidated, including the topotactic dehydration, valence and spins exchange mechanisms suggestion and the formation of a mixture of CoFe_2_O_4_-Co_3_O_4_ (3:1) as final products of thermal degradation.

**Results:**

The course of thermal decomposition of **1a** in air atmosphere up to 600°C was monitored by TG/DSC techniques, ^57^Fe Mössbauer and IR spectroscopy. As first, the topotactic dehydration of **1a** to the hemihydrate [Co(*en*)_3_][Fe(CN)_6_] ∙∙ 1/2H_2_O (**1b**) occurred with preserving the single-crystal character as was confirmed by the X-ray diffraction analysis. The consequent thermal decomposition proceeded in further four stages including intermediates varying in valence and spin states of both transition metal ions in their structures, i.e. [Fe^II^(en)_2_(μ-NC)Co^III^(CN)_4_], Fe^III^(NH_2_CH_2_CH_3_)_2_(μ-NC)_2_Co^II^(CN)_3_] and Fe^III^[Co^II^(CN)_5_], which were suggested mainly from ^57^Fe Mössbauer, IR spectral and elemental analyses data. Thermal decomposition was completed at 400°C when superparamagnetic phases of CoFe_2_O_4_ and Co_3_O_4_ in the molar ratio of 3:1 were formed. During further temperature increase (450 and 600°C), the ongoing crystallization process gave a new ferromagnetic phase attributed to the CoFe_2_O_4_-Co_3_O_4_ nanocomposite particles. Their formation was confirmed by XRD and TEM analyses. In-field (5 K / 5 T) Mössbauer spectrum revealed canting of Fe(III) spin in almost fully inverse spinel structure of CoFe_2_O_4_.

**Conclusions:**

It has been found that the thermal decomposition of [Co(*en*)_3_][Fe(CN)_6_] ∙∙ 2H_2_O in air atmosphere is a gradual multiple process accompanied by the formation of intermediates with different composition, stereochemistry, oxidation as well as spin states of both the central transition metals. The decomposition is finished above 400°C and the ongoing heating to 600°C results in the formation of CoFe_2_O_4_-Co_3_O_4_ nanocomposite particles as the final decomposition product.

## Background

Transition metal hexacyanido complexes represent an enormous and well-known group of coordination species. The Prussian blue, with the idealized formula Fe_4_[Fe(CN)_6_]_3_ · *n*H_2_O, is one of the oldest examples of a hexacyanido complex combining interesting structural features and magnetic properties. Therefore, many Prussian blue analogues [1], e.g. M_x_A[B(CN)_6_]_z_ · *n*H_2_O (M is alkali metal, A and B are different divalent/trivalent transition metals, x ≤ 1, z ≤ 1, if z < 1 B-sites are fractionally occupied and B-vacancies are present) have been extensively studied due to their sorption, ion-exchange, electron transfer or magnetic properties, and in recent years, many applications using PBAs usually in form of thin films or nanoparticles have been developed [2,3].

The reversible intercalation of alkali metal cations due to the zeolite-like structure of PBAs [4] has been employed in still active development of the selective adsorbent of ^137^Cs from radioactive waste waters [5] or more recently, in construction of a new active electrode material for Li-ion batteries (A_x_Mn_y_[Fe(CN)_6_] where A = K, Rb, x < 1, 1 < y < 1.5) [6]. The electrochemical coating of electrodes with thin film of PBAs or deposition with coordinated PBAs nanoparticles [2,7,8] gave rise a large number of amperometric and potentiometric sensors for non-electroactive ions or H_2_O_2_[3,9], electrochromic sensors [10] or biosensors [11]. The electrochromic or thermochromic behavior of electrochemically prepared PBAs thin films was further applied in the construction of different devices including electrochromic displays and pattering devices, thermal probes or photoelectrochemical/photocatalytical solar energy convertors [12].

PBAs also represent an enormous and important group of molecular magnets [1,13-15] which play important roles in many applications mainly in molecular electronics and magnetic/electronic devices [16,17]. During the experimental race to enhance the Curie temperatures of molecular magnets based on PBAs, the systems incorporating V(II) gave the most promising results and KV[Cr(CN)_6_]**·**2H_2_O was found to possess the highest *T*_c_ even above the boiling point of water (376 K) [18]. This invention of room temperature molecule-based magnets opens interesting applications as magnetic switches, oscillators or magneto-optical data storage devices. The cyanido-bridged networks, serving as a platform for the preparation of coordination nanoparticles based on Prussian blue, has also been employed for the controlling/tuning of magnetism by temperature (e.g. A^I^[M^II^M^III^(CN)_6_], A = alkali metal ion) [2] or light irradiation (with a general formula of A_*x*_Co_*y*_[Fe(CN)_6_]_*z*_ · *n*H_2_O; x, y, z are independent) [8].

An important feature of especially Co-Fe Prussian blue systems is the phenomenon of photoinduced magnetization or photoreversible enhancement of magnetization [19-21] which has been observed in e.g. Rb_1.8_Co^III^_3.3_Co^II^_0.7_[Fe^II^(CN)_6_]_3.3_ · 13H_2_O [22,23], Na_0.34_Co_1.33_[Fe(CN)_6_] · 4.5H_2_O [19], or K_0.4_Co_1.4_[Fe(CN)_6_] · 5H_2_O [20]. These compounds are efficient 3D-ferrimagnets at low temperatures (*T*_c_ < 30 K) due to the presence of majority of Fe^II^–CN–Co^III^ diamagnetic pairs which undergo upon irradiation photoinduced electron transfer resulting in paramagnetic Fe^III^–CN–Co^II^ species. Such transformation is accompanied by elongation of the Co–N bond lengths (0.15–0.20 Å) which is possible only in the case of flexible inorganic network provided by the existence of hexacyanidoferrate vacancies occupied by water molecules.

Transition metal hexacyanido complexes have been also successfully utilized as building blocks for constructing oligo- and polynuclear assemblies derived from the Prussian blue and a large number of such organic–inorganic hybrid materials containing various organic ligands, mainly *N*-donor linear or macrocyclic ligands, have been extensively studied [24-28]. These compounds were also found to behave as molecular magnets [29] with potential applications as molecular devices or memory devices. Some of them also revealed very specific optical properties including ion pair charge-transfer (IPCT) [30] transitions corresponding to the transfer of electrons from the complex anions to the complex cations in the visible and near-ultraviolet spectral regions. This phenomenon was confirmed for ionic pair compounds involving both [Co(*en*)_3_]^3+^ and [M(CN)_x_]^4–^ (for x = 6, M = Fe, Ru, Os; for x = 8, M = Mo, W) complex ions [31]. An irradiation of solutions containing these species into the IPCT absorption band (λ_irr_ = 405 or 510 nm) leads to a structure re-arrangement and the formation of bi- or trinuclear species. For the ionic pairs [M(NH_3_)_5_L]^3+^ and [Fe(CN)_6_]^3–^ (M = Ru, Os; L = NH_3_ or H_2_O), an inverse direction of the electron transfer of the IPCT transition (from the cation to the anion) was found [32].

Only eleven X-ray structures of discrete dinuclear Co-Fecyanido-bridged complexes have been reported up to now, whereas about ninety hits corresponding to poly- or oligomeric Co-Fe cyanido-bridged complexes have been deposited within the Cambridge Structural Database up to now [33]. Most of the dinuclear complexes have the general formula of [*L*_*n*_Co^III^–NC–Fe^II^(CN)_5_]^–^, where *L*_n_ stands for a macrocyclic ligand, and they have been systematically studied by Bernhardt *et al.*[34-36]. Up to date, similar number of ionic pair Co(III)-Fe(III) complexes have been structurally characterized. They always contain hexacyanidoferrate(III) anion and Co(III) complexed by ammonia [37], *en*[38-41], *bipy*[42-45], *terpy*[46], or other suitable ligands [47]. Despite Co(III)-Fe(III) hexacyanidoferrates represent very important group of compounds bearing a broad spectrum of important physico-chemical properties, surprisingly only a little attention has been paid to the monitoring of the thermal behavior of these compounds [48,49]. Especially the fact that they can serve as suitable precursors for thermally induced solid-state synthesis of magnetic nanoparticles [50,51] is almost unexplored.

The dehydration process of the monohydrate [Co(*en*)_3_][Fe(CN)_6_]∙ H_2_O was previously described in details by our working group [40] and recently, thermal decomposition of [Co(*en*)_3_][Fe(CN)_6_]∙ 2H_2_O in different atmospheres has been published as well [52]. In the present work, we also report the thermal degradation of [Co(*en*)_3_][Fe(CN)_6_]∙2H_2_O (**1a**) which was obtained upon recrystallization from water at 60°C. But furthermore, we concentrate on the progress of thermal decomposition of [Co(*en*)_3_][Fe(CN)_6_]∙2H_2_O under dynamic heating in air which includes the topotactic [53] dehydration process of the starting complex and gradual ligand liberation/decomposition resulting in the formation of CoFe_2_O_4_-Co_3_O_4_ nanocomposite particles as a final product of the thermal conversion. Based on the elemental and TG/DSC analyses, IR and ^57^Fe Mössbauer spectra, we suggest and discuss the unique decomposition mechanism accompanied by changes in the valence and spin states of both transition metal ions.

## Results and discussion

### Synthesis, X-ray structure and spectral characterization of [Co(*en*)_3_][Fe(CN)_6_]· 2H_2_O (1a)

In contrast to our previous study which described a topotactic dehydration process of [Co(*en*)_3_][Fe(CN)_6_]∙ H_2_O into [Co(*en*)_3_][Fe(CN)_6_] by means of single crystal X-ray analysis and Mössbauer spectroscopy [40], herein, we strive to elucidate the mechanism of the thermal decomposition of [Co(*en*)_3_][Fe(CN)_6_]∙ 2H_2_O based on a complete characterization of the decomposition intermediates. The ionic pair [Co(*en*)_3_][Fe(CN)_6_]∙ 2H_2_O complex was prepared by the straightforward reaction between [Co(*en*)_3_]^3+^ and [Fe(CN)_6_]^3–^, used as building blocks, in DMF [38]. The efforts to prepare the title compound by an one-pot redox reaction in water using CoCl_2_⋅ 6H_2_O instead of [Co(*en*)_3_]Cl_3_∙ 3H_2_O were unsuccessful probably due to the incomplete coordination of *en* and formation of Co(II)-involving PBA [23]. The synthesis following the Bok’s published procedure led to the formation of the dihydrated complex, however, two products, i.e. the dihydrated and monohydrated complexes, can be obtained after the product recrystallization from hot water in dependence on the rate of cooling of the solution. Concretely, the monohydrate is formed in the case of fast cooling of the solution heated up to 90°C, while the dihydrate is formed in the case of slow cooling of the 60°C warm solution.

**Figure 1:**
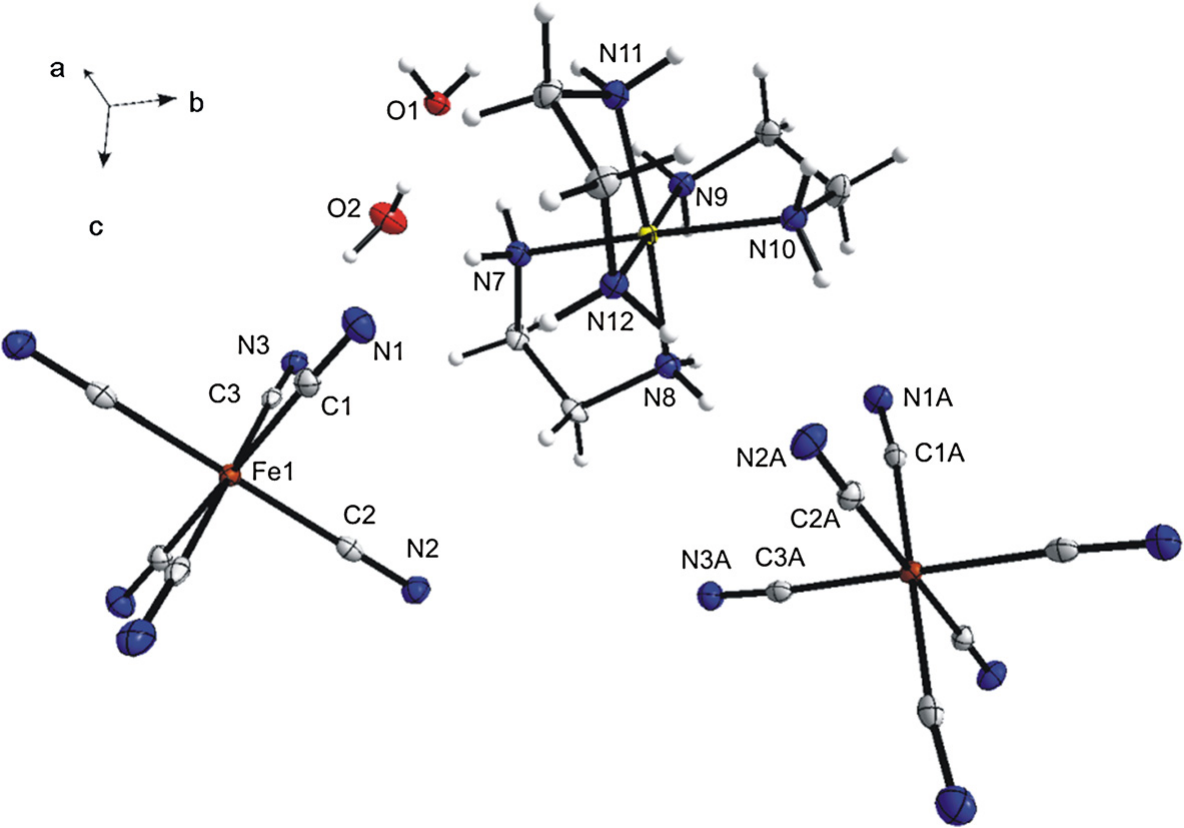
**Molecular structure of [Co( *en *)**_**3**_**][Fe(CN)**_**6**_**]⋅ 2H**_**2**_**O (1a).** The thermal ellipsoids are drawn at the 50% probability level.

As first, the molecular and crystal structures of **1a** have been determined by using single crystal X-ray analysis (Figure 1, Table 1). The crystallographically independent part of the molecule consists of the [Co(*en*)_3_]^3+^ cation, two halves of the [Fe(CN)_6_]^3–^ moiety with the iron(III) atoms lying on special positions (inversion centers) and two crystal water molecules (Figure 1). As the structure posses an inversion center, both *Λδδδ (lel*_*3*_*)* and *Λλλλ (ob*_*3*_*)* conformations of the [Co(*en*)_3_]^3+^ cations are present [54]. The Co(III) atom is coordinated by six nitrogen atoms from three chelating molecules of *en* in a distorted octahedral geometry. The Co–N bond lengths ranges from 1.955(2) to 1.981(2) Å (Table 2), and surprisingly, these values are significantly shorter than those determined for the same structure by Bok [1.996(3)–2.035(4) Å] [38]. For comparison, very similar values were found for [Co(*en*)_3_]I_3_ · H_2_O (1.950(4)–1.981(4) Å) [55] and for [Co(*en*)_3_]Cl_3_ (1.967(11) Å) [56], while they are much more distributed in [Co(*en*)_3_]_3_[FeCl_6_]Cl_6_**·**H_2_O (1.919(5)–2.002(5) Å) [57]. The differences may be caused by the counter-ion that significantly influences non-bonding interactions within the crystal structure and thus, a distortion in the vicinity of the central Co(III) atom can be found as a consequence of these interactions.

**Table 1:** Crystal data and structure refinements for 1a and 1b

	**1a**	**1b**
Formula	C_12_H_28_CoFeN_12_O_2_	C_24_H_50_Co_2_Fe_2_N_24_O
Formula weight	487.24	920.44
Crystal system	monoclinic	monoclinic
Space group	*P*2_1_/n	*P*2_1_/c
Unit cell dimension:		
*a* [Å]	8.2822(2)	15.0448(3)
*b* [Å]	16.5364(3)	8.4631(2)
*c* [Å]	14.5365(3)	14.9812(3)
*β* [°]	91.988(2)	90.160(2)
*Z*	4	2
*V*[Å^3^]	1989.69(7)	1907.48(7)
*D*_calc._ [g.cm^–3^]	1.627	1.603
*T* [K]	100	100
*μ* [mm^–1^]	1.599	1.658
Index ranges	−9 ≤ *h* ≤ 9, –16 ≤ *k* ≤ 19	−17 ≤ *h* ≤ 17, –8 ≤ k ≤ 10
	−17 ≤ *l* ≤ 17	−17 ≤ l ≤ 17
*θ*-range for data collection	2.75–25.10	2.71–25.0
Reflection collected	13225	9451
Independent reflections	3533 [*R*_int_ = 0.0286]	3348 [*R*_int_ = 0.0164]
Data/restrains/parameters	3533 / 4 / 250	3348 / 2 / 247
Goodness-of-fit on *F*^2^	1.169	1.246
Final R indices	*R*_1_ = 0.0265, *wR*_2_ = 0.0807	*R*_1_ = 0.0263, *wR*_2_ = 0.0796
R indices (all data)	*R*_1_ = 0.0354, *wR*_2_ = 0.0976	*R*_1_ = 0.0333, *wR*_2_ = 0.0949
Largest diff. peak and hole	0.444 and −0.424	0.465 and −0.627

**Table 2:** Selected bond lengths (Å) and angles (°) for 1a and 1b

**1a**		**1b**	
Co1–N7	1.955(2)	Co1–N4	1.964(2)
Co1–N8	1.977(2)	Co1–N5	1.963(2)
Co1–N9	1.978(2)	Co1–N6	1.966(2)
Co1–N10	1.981(2)	Co1–N7	1.956(2)
Co1–N11	1.971(2)	Co1–N8	1.968(2)
Co1–N12	1.968(2)	Co1–N9	1.966(2)
Fe1A–C1A	1.941(3)	Fe1A–C1A	1.947(3)
Fe1A–C2A	1.932(3)	Fe1A–C2A	1.927(3)
Fe1A–C3A	1.946(3)	Fe1A–C3A	1.944(3)
Fe1–C1	1.951(3)	Fe1–C1	1.941(3)
Fe1–C2	1.948(3)	Fe1–C2	1.946(3)
Fe1–C3	1.944(3)	Fe1–C3	1.928(3)
N7–Co–N8	85.59(9)	N4–Co–N5	85.40(9)
N9–Co–N10	84.82(9)	N6–Co–N7	85.74(9)
N11–Co–N12	85.67(9)	N9–Co–N8	85.55(9)

In the crystal structure, the Fe(III) atoms are coordinated by six carbon atoms originating from terminal cyanide groups. While the {Fe1C_6_} octahedron is almost regular (the Fe–C distances range from 1.944(3) to 1.951(3) Å), the {Fe1AC_6_} octahedron is slightly compressed with the shorter axial Fe1A–C2A distances (1.932(3) Å) as compared to the equatorial ones (1.941(3) and 1.946(3) Å) (Table 2). Such type of deformation of the hexacyanidoferrate(III) anions was already observed, e.g. in [(*aed*)_2_Cu_2_ClFe(CN)_6_]_n_ · 2H_2_O, where the Fe–C bond lengths range from 1.931(4) to 1.953(4) Å [58]. The remaining geometric parameters determined for [Fe(CN)_6_]^3–^ in **1a** are comparable to those found for the same complex anions in other compounds, e.g. in [Ni(*tren*)]_3_[Fe(CN)_6_]_2_·6H_2_O [59] or [Ni(*en*)_2_]_3_[Fe(CN)_6_]_2_·2H_2_O [60].

**Figure 2:**
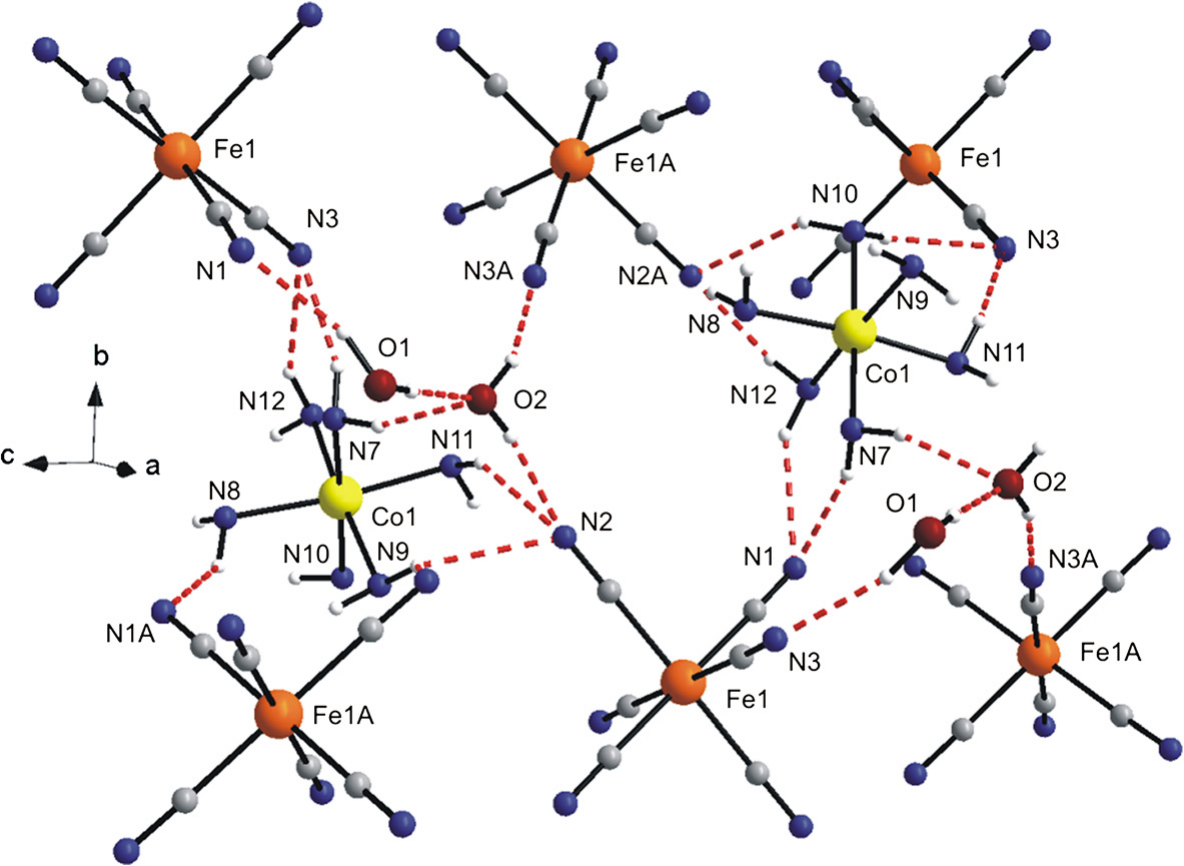
**Part of the crystal structure of [Co(*en)***_**3**_**][Fe(CN)**_**6**_**]⋅ 2H**_**2**_**O (1a) showing selected non-covalent contacts (dashed lines).** The CH_2_ groups of the ethylenediamine ligands are omitted for clarity. The thermal ellipsoids are drawn at the 50% probability level.

The secondary structure of **1a** is further stabilized by the O–H⋯O, O–H⋯N and N–H⋯N hydrogen bonds together with the C–H⋯N and N–H⋯C non-covalent contacts connecting the complex ions and crystal water molecules (Figure 2, Table 3).

**Table 3:** Selected hydrogen bonds for 1a and 1b (Å, °)

**D–H· · · A**	**d(D–H)**	**d(H· · · A)**	**∠ DHA**	**d(D· · · A)**
**1a**				
O1–H1V· · · O2	0.92	1.99	176.8	2.905(2)
O1–H1W· · · N3	1.00	2.08	163.8	3.050(3)
O2–H2V· · · N3A^i^	1.01	1.83	161.6	2.807(3)
O2–H2W· · · N2^i^	0.77	2.12	163.9	2.862(3)
N7–H7D· · · O2	0.92	2.00	159.2	2.882(3)
N7–H7C· · · N1	0.92	2.08	159.1	2.960(3)
N8–H8C· · · N1A^iii^	0.92	2.35	136.8	3.083(3)
N9–H9D· · · N2A^iii^	0.92	2.55	130.3	3.227(3)
N9–H9D· · · N2^i^	0.92	2.44	140.6	3.205(3)
N10–H10C· · · N3^ii^	0.92	2.35	153.2	3.203(3)
N10–H10D· · · N2A	0.92	2.42	145.5	3.226(3)
N11–H11C· · · N3^ii^	0.92	2.15	158.0	3.019(3)
N11–H11D· · · N2^i^	0.92	2.35	145.3	3.153(3)
N12–H12C· · · N1	0.92	2.39	151.0	3.227(3)
N12–H12D· · · N2A	0.92	2.08	165.3	2.976(3)
**1b**				
O1–H1W· · · N3^i^	1.20	1.67	139.3	2.695(7)
N4–H4C· · · N2A^iii^	0.92	2.12	159.4	2.997(3)
N5–H5C· · · N1A^iv^	0.92	2.20	151.4	3.039(3)
N5–H5D· · · O1	0.92	1.93	146.7	2.742(7)
N6–H6C· · · N1	0.92	2.32	143.1	3.108(3)
N6–H6D· · · N1A	0.92	2.43	137.1	3.168(3)
N7–H7C· · · N2A^iii^	0.92	2.19	145.9	2.996(3)
N7–H7D· · · N3^i^	0.92	2.20	156.9	3.069(4)
N8–H8D· · · N3	0.92	2.19	148.0	3.015(4)
N9–H9C· · · N1^v^	0.92	2.31	143.7	3.102(3)
N9–H9D· · · N2^ii^	0.92	2.06	148.8	2.887(3)

Symmetry codes (**1a**): i: x + 1/2, –y + 1/2, z–1/2; ii: x–1/2, –y + 1/2, z–1/2; iii: x + 1/2, y, z.

Symmetry codes (**1b**): i: x, y + 1, z; ii: x, –y + 1/2, z + 1/; iii: –x + 1, –y + 1, –z; iv: –x + 1, y + 1/2, –z + 1/2; v: –x + 2, –y + 1, –z.

**Figure 3:**
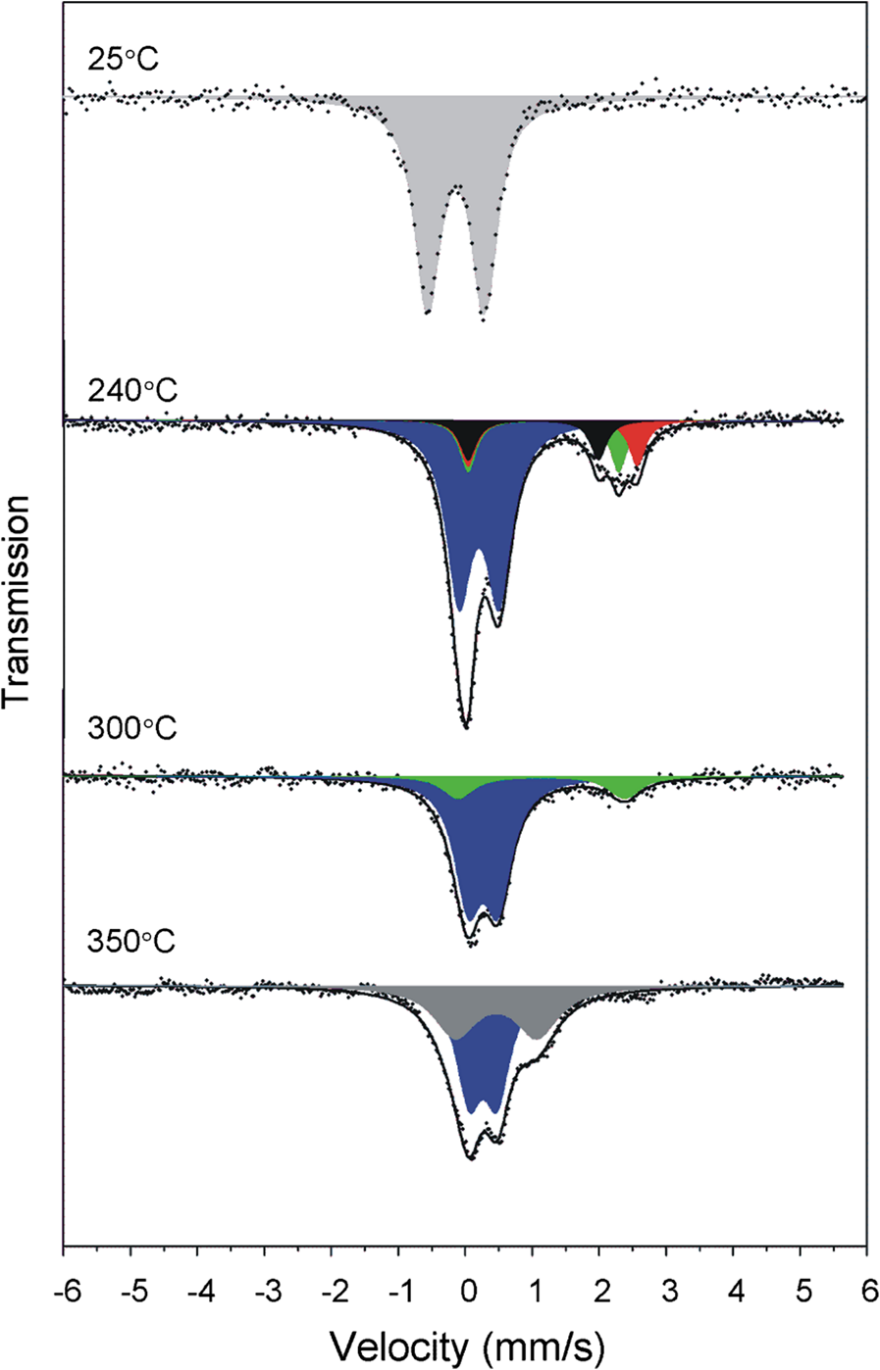
**A representative Mössbauer spectrum of [Co(*en*)**_**3**_**][Fe(CN)**_**6**_**]⋅ 2H**_**2**_**O (1a) at 25°C and spectra of the converted intermediates formed by heating of 1a up to 240, 300 and 350°C.**

**Figure 4:**
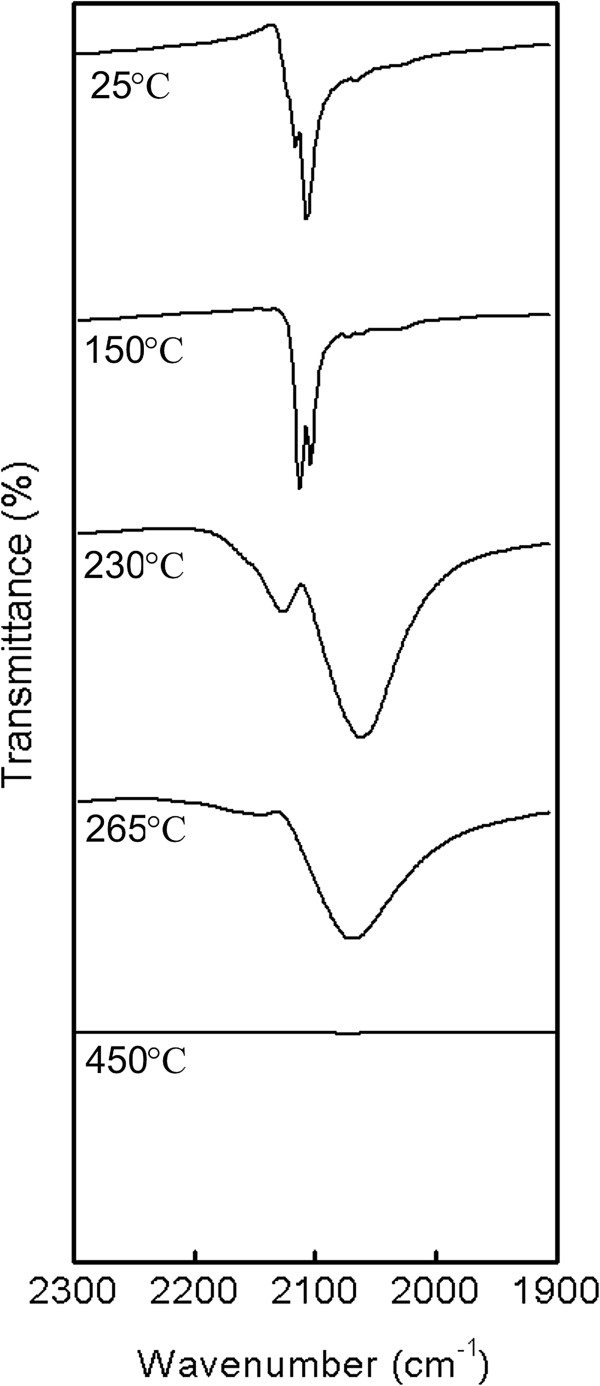
**Part of the room temperature IR spectrum of [Co(*en*)**_**3**_**][Fe(CN)**_**6**_**]⋅ 2H**_**2**_**O (1a, 25°C) and its comparison with the IR spectra of the corresponding decomposition intermediates at the temperatures of 150, 230, 265 and 450°C.**

The information on the local electronic state of the iron atom can be quantitatively obtained from ^57^Fe Mössbauer spectra. Concretely, the isomer shift, *δ*, which is related to the s-electron density at the nucleus and quadrupole splitting, Δ, related to the electric field gradient, are crucial parameters sensitive to the oxidation and spin states of iron atom as well as its coordination number. Thus, room temperature Mössbauer spectrum of **1a** confirmed the presence of Fe(III) ions in low-spin state because it consists a symmetrical doublet with isomer shift δ = −0.17 mm/s and quadrupole splitting ΔE_Q_ = 0.85 mm/s (Figure 3). The values of these parameters are typical for the hexacyanidoferrate(III) anion [61]. In measured IR spectra, typical sharp and strong absorption bands at 2117 and 2107 cm^–1^ corresponding to the *υ*(C≡N) stretching vibration were identified (Figure 4). They well document the presence of terminal coordinated cyanide groups because the vibration frequency of free CN^–^ anion (about 2080 cm^–1^) is blue shifted due to a coordination (removing electrons from weakly antibonding 5σ orbital during metal-carbon σ bond formation) like in K_3_[Fe(CN)_6_] which displays one sharp absorption band at 2118 cm^–1^[41,62]. In comparison with K_3_[Fe(CN)_6_], there is a splitting of the absorption band that can be explained by lowering of the *O*_h_ site symmetry of the hexacyanidoferrate(III) anion. Otherwise, broad absorption bands due to the O–H (~3380 cm^–1^), N–H (~3234 cm^–1^) or C–H (~3095 cm^–1^) stretching as well as deformation vibrations (NH_2_, 1630–1578 cm^–1^) are present together with the pattern of fingerprint region corresponding to the three coordinated molecules of *en* (the full IR spectrum is available in Additional file 1: Figure S1).

The temperature dependence of the effective magnetic moment (for details see Additional file 1: Figure S2) revealed weak antiferromagnetic interactions. The effective magnetic moment of **1a** at room temperature was 2.23 B.M. and it is gradually decreased to 1.84 B.M. as the temperature was reduced to 2 K. Its value was higher than expected for the spin-only value of 1.73 B.M. for *S* = 1/2, but similar to the value of 2.25 B.M. obtained for K_3_[Fe(CN)_6_] [63]. Fitting of the reciprocal susceptibility (see Additional file 1: Figure S3) above 50 K by Curie-Weiss law gave *g* = 2.67 and Θ = −19 K for *S* = 0 and *S* = 1/2 for Co^III^, and Fe^III^, respectively. The higher values of *g*-factors have been previously observed for other low-spin Fe^III^ complexes as well [64].

### Thermal behavior of [Co(*en*)_3_][Fe(CN)_6_]∙ 2H_2_O (1a) – a general overview

**Figure 5:**
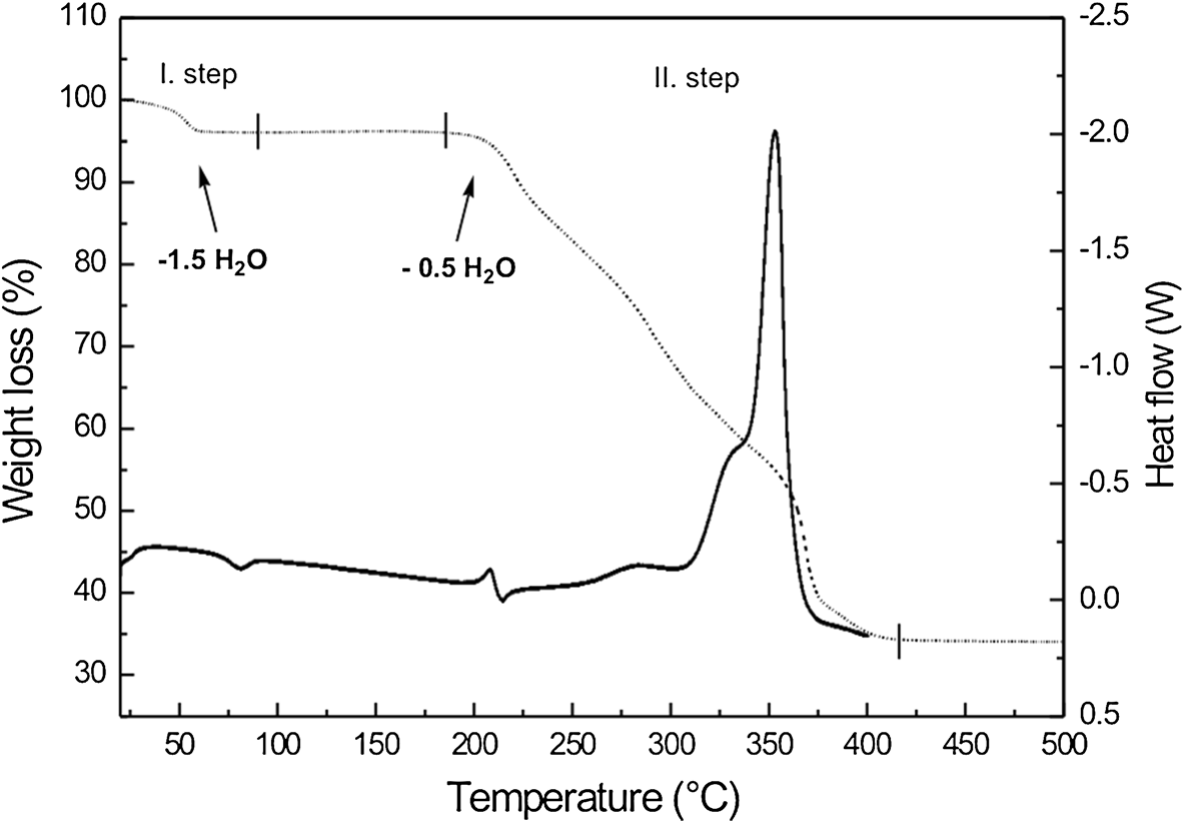
**TG (dotted) and DSC (full) curves of complex [Co(*en*)**_**3**_**][Fe(CN)**_**6**_**]⋅ 2H**_**2**_**O (1a).** Data were recorded in the temperature interval of 20–500, and 20–400°C, respectively, and with the heating rate of 5°C/min.

The TG and DSC curves of **1a** are displayed in Figure 5. TG curve of **1a** reveals two separable steps of the thermal decomposition. The first weight loss occurs in the temperature range of 50–80°C and it is accompanied by endothermic effect with the minimum at 80°C, as can be seen on DSC curve (Figure 5). Experimental weight loss (5.6%) is in good agreement with the calculated one (5.5%) corresponding to the release of 1.5 crystal water molecules from the formula unit of **1a** and thus, yielding the complex [Co(*en*)_3_][Fe(CN)_6_]**∙ **1/2H_2_O (**1b**), as confirmed by chemical and single crystal X-ray analyses. The hemihydrate **1b** is thermally stable within the temperature interval of 72–187°C. The second step of the thermal decay is observed in the temperature range of 187–420°C, and involves continual and complete sample decomposition. Based on the course of DSC curve, it can be stated that at least three processes take place: (i) a weak exothermic process with the maximum at 208°C, (ii) an endothermic process with the minimum at 214°C and (iii) a strong exothermic effect with the maximum at 354°C. The sample weight loss corresponding to the second step was found to be 61.7%. It can be assigned to elimination of the remaining water content, all molecules of ethylenediamine as well as all CN^–^ ligands and/or oxidation of the transition metal ions yielding a spinel-type phase with the general chemical composition of Co_1.5_Fe_1.5_O_4_ (calculated weight loss 62.1%). In comparison with the thermal decomposition of **1a** recently published in Ref. [52], the decomposition of **1a** occurs at lower temperature in our case, which can be associated to different experimental conditions, and moreover, CoFe_2_O_4_ only was determined as the final decomposition product [56]. Unstable intermediates [Fe^II^(*en*)_2_(μ-NC)Co^III^(CN)_5_], [Fe^III^(H_2_NCH_2_CH_3_)_2_(μ-NC)_2_Co^II^(CN)_5_] and Fe^III^[Co^II^(CN)_5_] were identified during this complicated decomposition. The detailed description of the above-mentioned processes with identification of the intermediates by XRD method, Mössbauer and IR spectroscopy and transmission electron microscopy (TEM) will be discussed below mainly from the chemical point of view (*vide infra*).

### Chemical, structural and spectral characterization of [Co(*en*)_3_][Fe(CN)_6_]∙**1/2H**_**2**_**O (1b) formed in the first decomposition step**

The complex [Co(*en*)_3_][Fe(CN)_6_]**∙ **1/2H_2_O (**1b**), as it was already mentioned, can be obtained as a stable intermediate by both dynamic or static heating of the dihydrate **1a** up to 130°C (Table 4). The empirical formula of **1b** was deduced from the elemental analysis results and well corresponds to the weight loss on the TG curve (Figure 5). The parameters of Mössbauer spectrum of **1b** (δ = −0.15 mm/s and ΔE_Q_ = 0.98 mm/s) exhibit small changes as compared to the corresponding values of **1a** (δ = −0.17 mm/s and ΔE_Q_ = 0.85 mm/s). Generally, the found hyperfine parameters refer to the presence of Fe(III) cations in the low-spin state in both complexes. Small increase in the quadrupole splitting value is evidently related to a small decrease in the symmetry of the iron atom surroundings as a consequence of the topotactic dehydration process. Similar findings, connecting with the variations in Mössbauer parameters depending on a number of crystal water molecules presented in the structure, were previously observed for a series of M_3_[Fe(CN)_6_]_2_ · *n*H_2_O complexes (M = Ni, Mn, Co, Cu, Cd, *n* can rise up to 14) [61]. There is only very small difference in measured IR spectra between **1a** and **1b**, because all above described absorption bands for **1a** are also present in the spectrum of **1b** (Figure 4).

**Table 4:** Room temperature Mössbauer parameters and the corresponding elemental analyses of complex 1a as well as the samples prepared by its dynamic heating up to 130–600°C

**Sample number**	**T [°C]**	**phase**	**δ [mm/s]**	**ΔE**_**Q **_**[mm/s]**	**H**_**hyp **_**[T]**	**RA [%]**	**Elemental analyses [%]**			**Assignment**
							**N**	**C**	**H**	
**1a**	25	LS Fe(III)	−0.17	0.85	–	100	34.4	29.8	5.5	[Co^III^(*en*)_3_][Fe^III^(CN)_6_]⋅ 2H_2_O
**1b**	130	LS Fe(III)	−0.15	0.98	–	100	36.0	31.2	5.4	[Co^III^(*en*)_3_][Fe^III^(CN)_6_]⋅ ½H_2_O
**2**	240	HS Fe(II)	1.17	2.25	–	13.0	31.3	33.2	3.9	[Fe^II^(*en*)_2_(μ-NC) Co^III^(CN)_4_]
		HS Fe(II)	1.31	2.53	–	11.4				
		HS Fe(II)	1.02	1.95	–	10.0				
		HS Fe(III)	0.22	0.60	–	65.6				[Fe^III^(NH_2_CH_2_CH_3_)_2_(μ-NC)_2_ Co^II^(CN)_3_]
**3**	300	HS Fe(II)	1.14	2.49	–	18.4	26.8	31.9	3.0	[Fe^II^(*en*)_2_(μ-NC) Co^III^(CN)_4_]
		HS Fe(III)	0.25	0.45	–	81.6				[Fe^III^(NH_2_CH_2_CH_3_)_2_(μ-NC)_2_ Co^II^(CN)_3_]
**4**	350	HS Fe(III)	0.26	0.43	–	55.7	19.9	22.8	2.0	[Fe^III^(NH_2_CH_2_CH_3_)_2_(μ-NC)_2_ Co^II^(CN)_3_]
		HS Fe(III)	0.46	1.22	–	44.3				Fe^III^[Co^II^(CN)_5_]
**5**	400	HS Fe(III)	0.31	0.76	–	100	0	0	0	CoFe_2_O_4_ (SP)
**6**	450	HS Fe(III)	0.31	0.82	–	46.8	0	0	0	CoFe_2_O_4_ (SP)
		HS Fe(III)	0.29	−0.01	42.6	53.2				CoFe_2_O_4_
**7**	600	HS Fe(III)	0.31	0.53	–	3.4	0	0	0	CoFe_2_O_4_ (SP)
		HS Fe(III)	0.29	−0.01	48.6	79.1				CoFe_2_O_4_
		HS Fe(III)	0.37	−0.05	51.4	17.5				CoFe_2_O_4_

(LS) low-spin state, (HS) high-spin state, (SP) superparamagnetic particles, (RA) relative abundance.

The single crystal character is preserved during the dehydration process of **1a** to **1b** what is, generally, a very rare phenomenon among coordination compounds although it is quite common for simple salts, e.g. K_0.35_CoO_2_· 0.4H_2_O [65], manganese(II) formate [66] or FePO_4_[67]. To our knowledge, the single crystal dehydration process was reported only for a series of polymeric compounds {[M(H_2_O)_2_(C_4_O_4_)(*bipy2*)]∙ H_2_O}_n_ (M = Co, Ni, Fe) [68], and [Ni(*en*)_3_]_3_[Fe(CN)_6_]_2_ · 2H_2_O [60] up to date.

The molecular structure of the hemihydrate **1b** (Figure 1, Tables 1 and 2) is analogous to that of the original dihydrate **1a**, since it is composed of the same complex cations and anions. Main, but not significant, structural variances between **1a** and **1b** are caused by the presence of different number of crystal water molecules. Thus, the dehydration process is accompanied by small shifts only in the positions of the respective [Co(*en*)_3_]^3+^ and [Fe(CN)_6_]^3–^ ions as can be deduced from the mean values of the Co⋯Fe distances to the six nearest neighbors which were found to be 6.32(4) and 6.22(4) Å in the crystal structures of **1a**, and **1b**, respectively. Both **1a** and **1b** crystallize in the monoclinic system in related space groups (i.e. *P*2_1_/n for the dihydrate and *P*2_1_/c for the hemihydrate), but with the different unit cell orientations towards the presented molecules. The relationship between two unit cell settings can be expressed by the equation (a_H_, b_H_, c_H_) = (010,100, 00–1) (a_D_, b_D_, c_D_), where, a_D_, b_D_, c_D_ and a_H_, b_H_, c_H_ stand for cell parameters of the hemihydrate (H), and dihydrate (D), respectively. It should be noted that the monoclinic angles in both unit cells [β_D_ = 91.982(4)° and β_H_ = 90.15(1)°] display only minor deviation from the right angle. As expected, the unit cell dimensions of the hemihydrate **1b** are somewhat shorter in lengths as compared to the dihydrate **1a**. Therefore the dehydration process from the dihydrate (**1a**) to the hemihydrate (**1b**) can be considered as topotactic because of minimal geometric changes (less than 4%) and minimal atomic and molecular movements (change of each intra-/intermolecular distances < 4.2 Å) [53].

**Figure 6:**
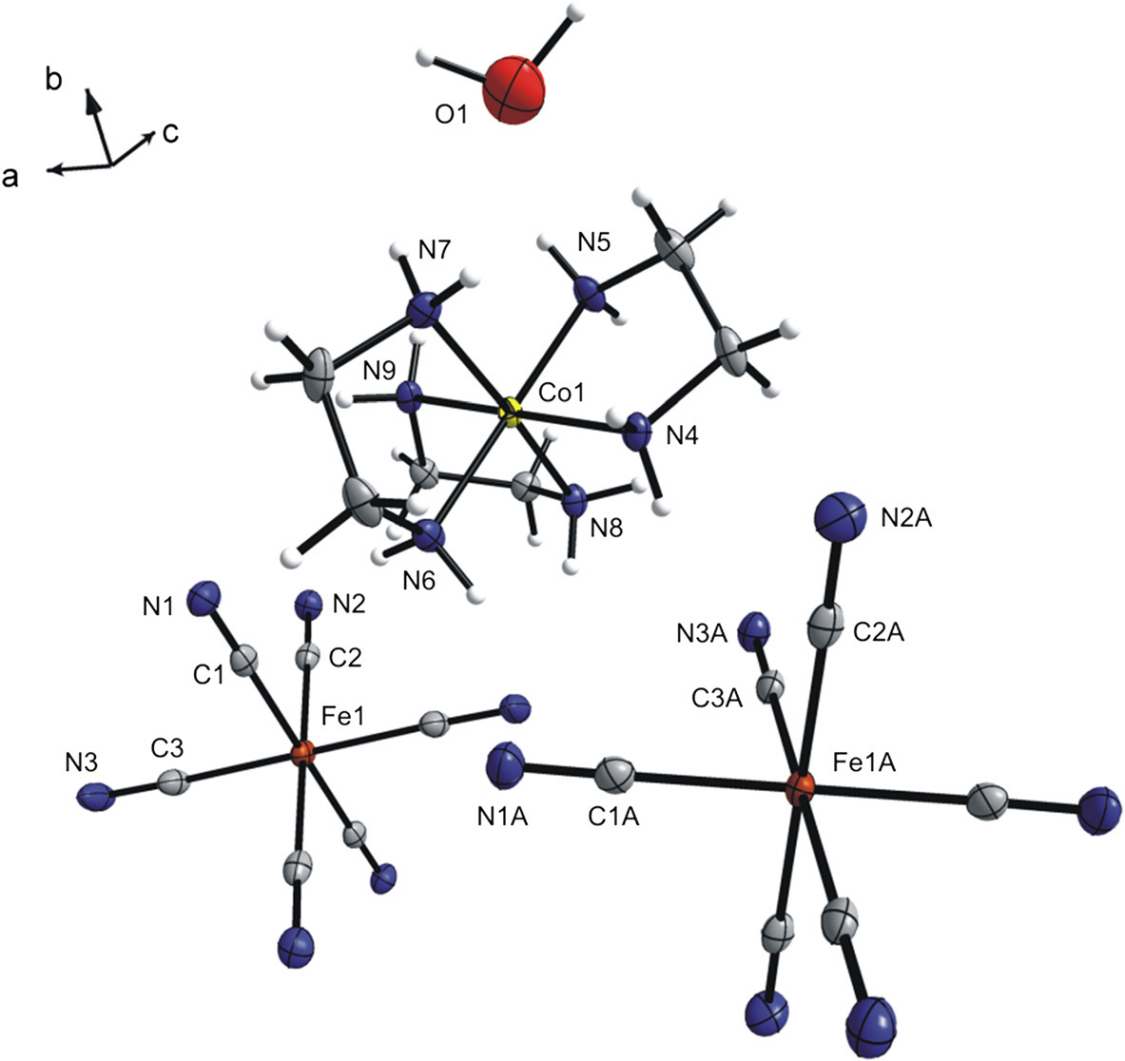
**Molecular structure of [Co(*en*)**_**3**_**][Fe(CN)**_**6**_**]⋅ 1/2H**_**2**_**O (1b).** The thermal ellipsoids are drawn at the 50% probability level.

Despite considerable similarity between both structures, also some differences can be found. The partial dehydration caused conformational change within the [Co(*en*)_3_]^3+^ cations. While the *Λδδδ* (and *Δλλλ*) conformation was observed in **1a**, the *Λλδδ* (and Δ*δλλ*) conformation was found in **1b** (Figure 6). It can be assumed that these conformational variations are induced by changes in the hydrogen bonding interactions (see below) as was previously suggested in conformation analysis of [M(*en*)_3_]^3+^ (M = Co^III^, Cr^III^) [54]. Moreover, together with the conformational changes within the [Co(*en*)_3_]^3+^ cations, the shortening in the Co–N bond lengths in the hemihydrate **1b** (1.956(2)–1.968(2) Å) were found as compared to those in **1a** (1.955(2)–1.981(2) Å). However, all these values are comparable to those determined for the same cation in similar systems (1.97(7) Å) [33].

Two crystallographically independent centrosymmetric [Fe(CN)_6_]^3–^ moieties (both iron(III) atoms occupy special positions – the inversion centers) are present in the structure of **1b**, similarly as in the structure of **1a**. Both Fe1C_6_ and Fe1AC_6_ octahedra in **1b** are slightly deformed. The octahedron around the Fe1A atom is compressed (Table 2) with the axial Fe1A–C2A distances (1.927(3) Å) being shorter than the equatorial ones (1.947(3) and 1.943(3) Å). Similar compression was found in the vicinity of the Fe1 atom with the axial Fe1–C3 distance (1.928(3) Å) and longer equatorial distances (1.946(3) and 1.941(3) Å). All these Fe–C bond lengths as well as other interatomic parameters are from the range of typical values for [Fe(CN)_6_]^3–^ anions.

**Figure 7:**
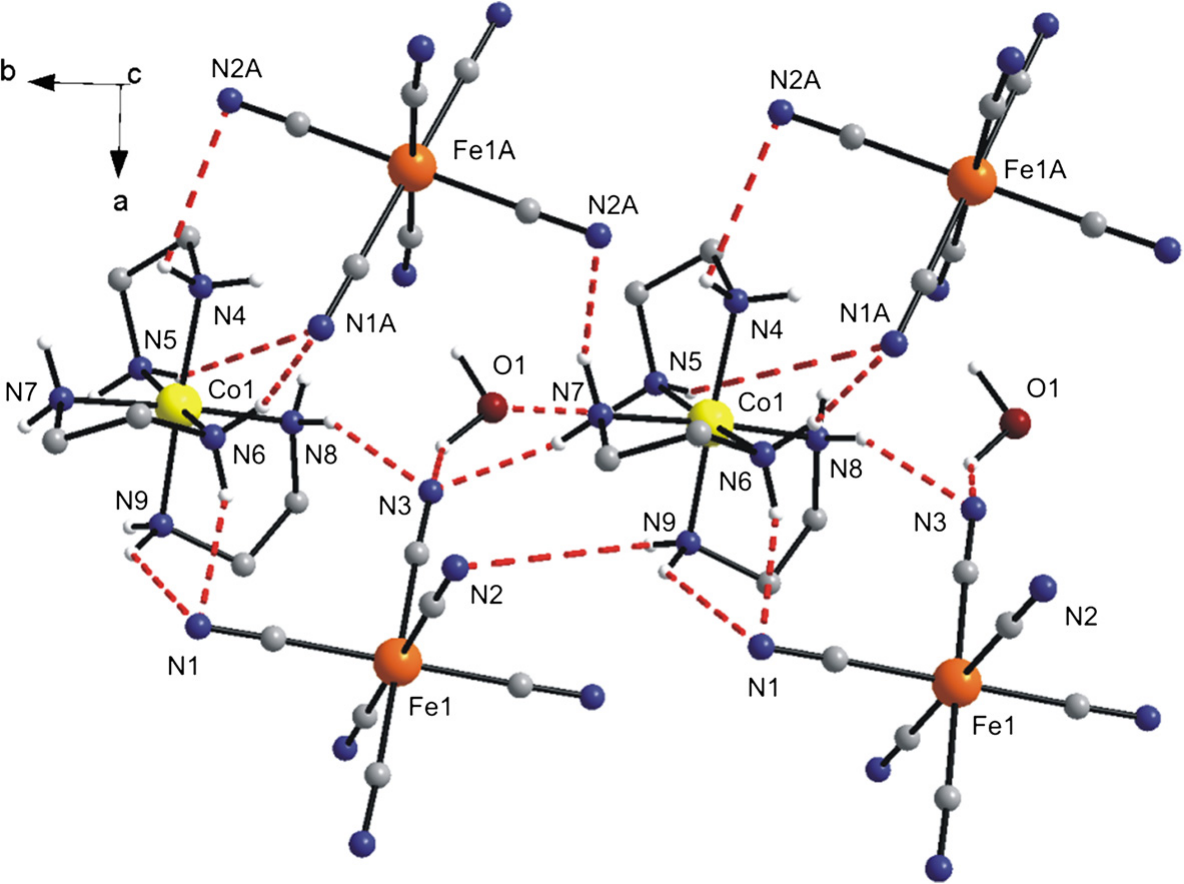
**Part of the crystal structure of [Co(*en*)**_**3**_**][Fe(CN)**_**6**_**]⋅ 1/2H**_**2**_**O (1b) showing selected non-covalent contacts (dashed lines).** Hydrogen atoms of the CH_2_ groups of the *en* ligands are omitted for clarity. The thermal ellipsoids are drawn at the 50% probability level.

Generally, it can be anticipated, that the topotactic dehydration may influence significantly an extent of hydrogen bonds and other non-covalent contacts presented within the crystal structures. Thus, as a consequence of this general statement, the number of the above-mentioned non-bonding interactions is slightly lower, and moreover, they are moderately shorter than those in the crystal structure of **1a**. While in the crystal structure of the dihydrate **1a,** the water molecules are connected with [Fe(CN)_6_]^3–^ anions via hydrogen bonds of the type O–H⋯N and O–H⋯O, the O–H⋯O hydrogen bond is absent in the crystal structure of **1b** (Table 3, Figure 7).

The magnetic properties of the hemihydrate **1b** (see Additional file 1: Figure S2 and S3) are similar compared to those for the dihydrate **1a**. Fitting of the temperature dependence of reciprocal susceptibility above 50 K by Curie-Weiss law gave *g* = 2.77 and *Θ* = −18 K indicating slightly weaker antiferromagnetic interaction in comparison with **1a**. The effective magnetic moment of **1b** at room temperature is similar to the value for **1a**, but a small difference becomes visible when the temperature declines below 50 K (Additional file 1: Figure S2) where the values of μ_eff_ for **1b** are slightly higher than those for **1a** and thus, indicating weaker antiferromagnetic interactions. Such observation is in accordance with the topotactic dehydration process which reduces the number of intra-molecular interactions as was observed in the crystal structure of **1b**. The completed CIF files for **1a** and **1b** can be found as Additional files 2, and 3, respectively.

### Chemical, structural and spectral characterization of the intermediates formed during the second and third decomposition steps

**Scheme 1:**
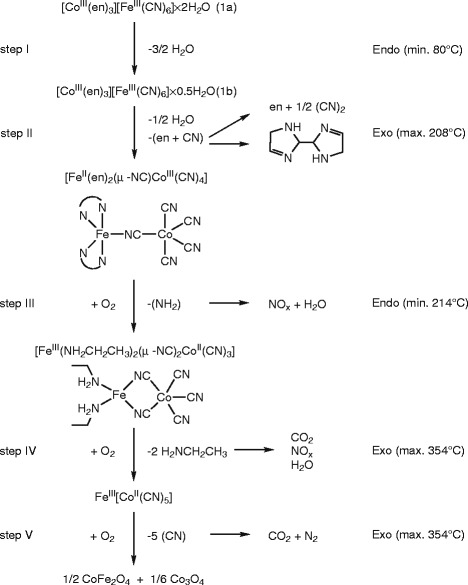
**Suggested scheme of thermal decomposition of [Co**^**III**^**(*en*)**_**3**_**][Fe**^**III**^**(CN)**_**6**_**]⋅ 2H**_**2**_**O (1a) during dynamic heating in air atmosphere.** (Endo stands for endo-effects, while Exo for exo-effects).

To explain the unexpected exothermic effects in DSC curves accompanying the evolution of molecules of *en* at higher temperatures and generally, to understand the structure and chemical composition of intermediates formed during the appropriate stage of the thermal treatment, the complex **1a** was heated dynamically in conditions of the DSC experiment up to the selected temperatures and the samples were analyzed consequently by Mössbauer and IR spectroscopy and elemental analysis. The results obtained from these measurements together with the proposed composition of conversion intermediates are summarized in Table 4. The suggested decomposition model for **1a** is depicted in Scheme 1.

When **1a** was heated up to 240°C (sample **2**), the color of sample turned from orange to green. It was demonstrated by DSC that this solid underwent an exo-effect with the maximum at 208°C followed by an endo-effect with the minimum at 214°C. The Mössbauer spectrum of **2** consists of four doublets which correspond to iron-containing species in two different oxidation states (see Figure 3 and Table 4). Three doublets with δ values ranging from 1.02 to 1.31 mm/s correspond clearly to the high spin Fe(II) atoms [69,70]. The high values of quadrupole splitting parameters (1.95–2.53 mm/s) manifest a very low symmetry of the Fe(II) environment as expected for the coordination number 5 [69]. The presence of three broadened Fe(II) doublets varying in the values of ΔE_Q_ is probably related to the various degrees of the deformation of the iron(II) environment. The most populated doublet (δ = 0.22 mm/s; ΔE_Q_ = 0.60 mm/s) can be ascribed to a high spin Fe(III) environment in tetrahedral coordination [70]. The increase of the isomer shift in comparison with **1a** or **1b** can be attributed to the iron spin transition from low to high spin as well as to the different coordination mode of cyanide ion to Fe(III) (Fe^III^–CN in **1a**,**b,** Fe^III^–NC in **2**) [50]. The results of Mössbauer spectroscopy give an evidence for the surprising change of the spin state of all iron atoms between 130 and 240°C as all the spectral components correspond to the high-spin states. Moreover, the presence of divalent iron indicates a central atom reduction which accompanies oxidation of CN^–^ to (CN)_2_. The formed cyanogen can react with two molecules of *en* which are also liberated during this thermolytic step and one bicyclic molecule of *bis*(Δ^2^-2-imidazolinyl) [71] could be formed as a potential product of the decomposition (see Scheme 1). All the mentioned experimental results can be explained by the formation of the bridging [Fe^II^(*en*)_2_(μ-NC)Co^III^(CN)_4_] complex from the hemihydrate **1b** (see step II in Scheme 1) what is in accordance with the preference of HS Fe(II) to be coordinated in weaker ligand field provided by N-donor atoms rather than C-donor atoms. It seems that the complete dehydration induces a drastic rearrangement of the environments of both central atoms related to the evolution of half molecule of cyanogen and one molecule of *en* from the formula unit of **1b**, which is probably reflected on the DSC curve by the mentioned exo-effect at 208°C. Nevetheless, similar process of deamination has been described previously on other systems containing [Co(*en*)_3_]^3+^ and it was always accompanied by a strong endo-effect above 200°C on the DSC curve [72]. Thus, the following endo-effect at 214°C on the DSC curve could be ascribed to the liberation of *en* as well.

The simultaneous presence of the high spin Fe(II) and Fe(III) species in sample **2** can be related to the partial transformation of [Fe^II^(*en*)_2_(μ-NC)Co^III^(CN)_4_] to [Fe^III^(NH_2_CH_2_CH_3_)_2_(μ-NC)_2_Co^II^(CN)_3_] (see step III in Scheme 1). This charge transfer process has been previously observed and confirmed in many Fe^II^–CN–Co^III^ species (light-induced magnetization changes) [19-23] and here, it is more pronounced at higher temperature as clearly seen from the comparison of the Fe(II)/ΣFe ratio of 0.34 found in sample **2** heated up to 240°C with that observed in sample **3** heated up to 300°C (0.18; see Table 4 and Figure 3). In this third decomposition step we assume the participation of atmospheric oxygen and gradual evolution of NO_X_. This assumption is in an accordance with the N/C ratio decrease between 240 (0.94) and 300°C (0.84; see Table 4). The step III is manifested in DSC curve through a long endo-effect with the minimum at 214°C or rather long exo-effect in the temperature range of 220–300°C without specified maximum (see Figure 5). It is worth to mention that both samples **2** and **3** revealed a drastic lowering of conductivity compared to hemihydrate **1b,** which is in line with the presence of bridging ligands between Co and Fe central atoms in the dinuclear species.

IR spectra recorded for the intermediates in sample **2** and **3** (230 and 265°C, Figure 4) confirmed proposed thermal decomposition mechanism. The *υ*(C≡N) stretching vibration is significantly governed by the electronegativity, oxidation state and coordination number of the metal central atom directly bonded to the cyanide group. Thus, the formation of cyanido-bridged species is accompanied by a significant broadening of the *υ*(C ≡ N) bands observed in IR spectra. Furthermore, the shift of the peak at 2113 cm^–1^ toward higher wave numbers with increasing temperature (2113 → 2127 → 2132 cm^–1^ for 150 → 230 → 265°C) documents the oxidation state changes and could be assigned to *υ*(Fe^II^–CN–Co^III^). The intensity of this vibration band decreases with higher temperature which indicates absence of such type of coordination above 265°C. On the other hand, the shift of the peak at 2104 cm^–1^ toward lower wave numbers with increasing temperature (2104 → 2062 → 2066 cm^–1^ for 150 → 230 → 265°C) corresponds to *υ*(Co^II^–CN–Fe^III^) [50]. This observation confirms the proposed decomposition mechanism concerning increasing amount of [Fe^III^(NH_2_CH_2_CH_3_)_2_(μ-NC)_2_Co^II^(CN)_3_] at higher temperature (see step III in Scheme 1 and Table 4) and it indicates that Co^II^–CN–Fe^III^ is the most probable coordination bridge in [Fe^III^(NH_2_CH_2_CH_3_)_2_(μ-NC)_2_Co^II^(CN)_3_]. Although non-linear coordination mode of bridging cyanide in di/polynuclear complexes is known, the suggested structure of [Fe^III^(NH_2_CH_2_CH_3_)_2_(μ-NC)_2_Co^II^(CN)_3_] requires two cyanide bridges of that type. Such coordination tension could be reduced if the formation of a structure with the cyclic tetranuclear {Fe_2_Co_2_(CN)_4_} core is considered. Moreover, compounds with similar core-structure have been described recently [73]. Another possibility is the formation of a polymeric structure with μ_3_-CN coordination mode found in the case of several Cu(I)–Zn(II) complexes.

### Chemical, structural and spectral characterizations of the intermediates formed in the fourth decomposition step

Although the samples **2** and **3** differ in quantitative characteristics, mainly in the C and N contents and in the percentage of Fe(II) and Fe(III) species, their phase composition is similar from the qualitative viewpoint as evident from hyperfine parameters of Mössbauer spectra. The next principal chemical change in the chemical composition of the samples takes place after heating up to 350°C (sample **4**). This fourth reaction step is reflected on the DSC curve at 335°C by a shoulder of exo-effect at 354°C and documented also by a considerable diminution of both C and N contents (see Table 4). In Mössbauer spectrum of the sample **4** (see Figure 3) heated up to 350°C, there are no indications of the divalent iron species in contrast to the room temperature spectra of samples **2** and **3**. On the other hand, a new high-spin Fe(III) phase with δ = 0.46 mm/s and quadrupole splitting ΔE_Q_ = 1.22 mm/s appears in the spectrum along with the residual doublet ascribed to [Fe^III^(NH_2_CH_2_CH_3_)_2_(μ-NC)_2_Co^II^(CN)_3_] as its hyperfine parameters are almost unchanged compared to the spectrum of sample **3**. The hyperfine parameters of the former species are in a very good accordance with those observed for iron complexes with pentacoordinated high spin Fe(III) environments [74]. Thus, this new phase with the low symmetry of iron environment, which is manifested by a high value of quadrupole splitting, can be ascribed to Fe^III^[Co^II^(CN)_5_]. As for the expected chemical nature of the gases evolved during the fourth decomposition step leading from [Fe^III^(NH_2_CH_2_CH_3_)_2_(μ-NC)_2_Co^II^(CN)_3_] to Fe^III^[Co^II^(CN)_5_] (see step IV in Scheme 1), their experimental identification using evolved gas analysis (EGA) may be ambiguous due to the negative role of atmospheric nitrogen and oxygen. Nevertheless, previous decomposition studies of other complexes with *en*, e.g. [Ni(*en*)_3_](*ox*) [75] using EGA showed that *en* is decomposed to a mixture of H_2_, CO/CO_2_, N_2_ and CH_2_ = CH_2_ in an inert atmosphere. Thus, the decomposition of ethylamine in air may involve similar or more oxygen containing species such as H_2_O, CO, CO_2_, N_2_ or NO_x_.

### Characterization of the final decomposition product – the fifth decomposition step

**Figure 8:**
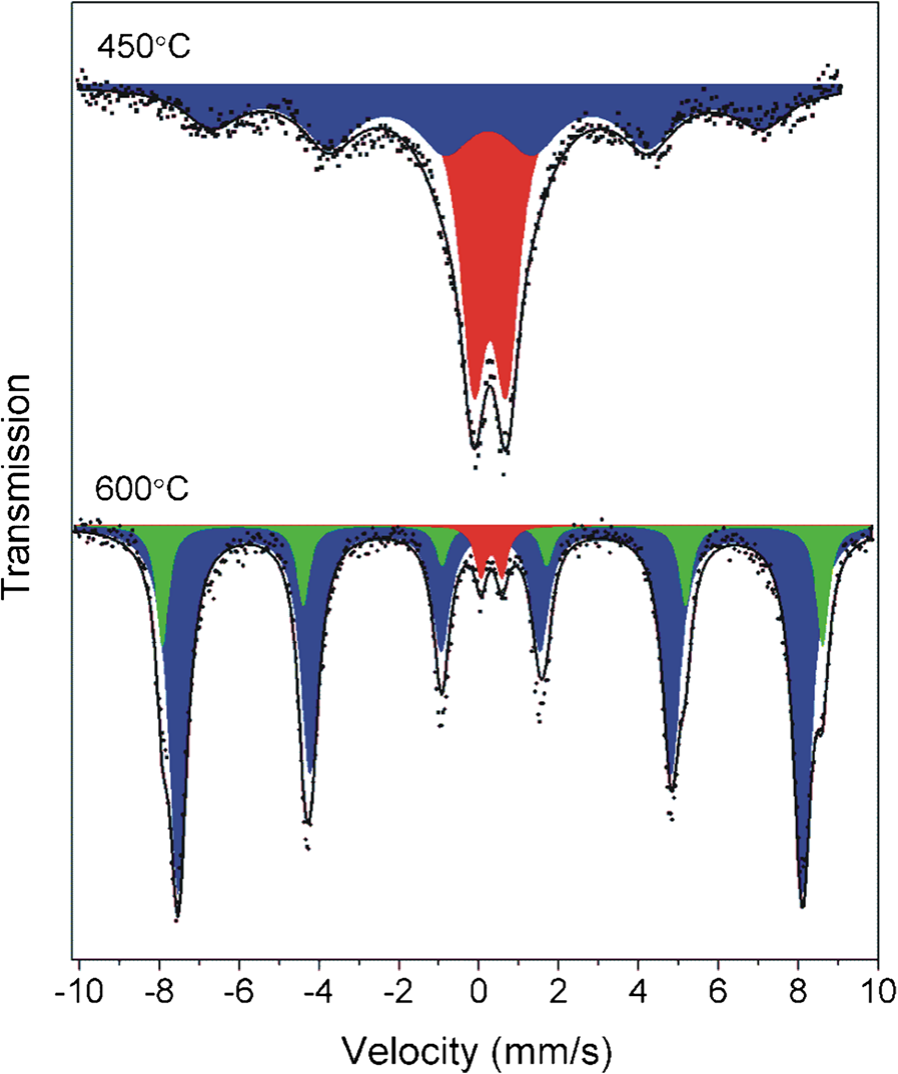
Room temperature Mössbauer spectra of the decomposition products prepared by dynamic heating of 1a up to 450°C (sample 6) and 600°C (sample 7).

In accordance with TG data, decomposition process is completed in dynamic conditions at 420°C. As mentioned above, the overall weight loss corresponds well with the final formation of the M_3_O_4_ (M = Fe, Co) phase(s) which is in accordance with the absence of *υ*(C ≡ N) stretching vibration bands in IR spectrum (Figure 4, 450°C). Taking into account the starting molar ratio of Fe/Co = 1/1, various combinations of several oxides (CoFe_2_O_4_, Fe_3_O_4_, Fe_2_O_3_ polymorphs, Co_3_O_4_, FeCo_2_O_4_) come on force to express the phase composition of the final product. The isomer shift values in room temperature Mössbauer spectra of the final decomposition products formed after dynamic heating of **1a** up to 450 and 600°C (samples **5**, **6** and **7**, see Table 4 and Figure 8) unambiguously exclude the presence of divalent iron (Fe_3_O_4_, FeCo_2_O_4_) in the iron oxide phase [76]. The spectra of the samples **5** and **6** heated up to 400 and 450°C display a broadened doublet as a prevailing component, which can be ascribed to superparamagnetic nanoparticles. Superparamagnetic character of the spectra disallows the identification of iron(III) oxide phase (CoFe_2_O_4_ vs. γ-Fe_2_O_3_ vs. α-Fe_2_O_3_) in all samples. In the spectrum of sample **6** heated to 450°C, the prevailing dublet is accompanied by a completely magnetically split sextet which becomes the major signal observed in the spectrum of sample **7** prepared by heating of **1a** up to 600°C (see Figure 8). This reduction of the ratio between the intensity of the dublet (superparamagnetic particles) and sextet manifests the formation of larger particles (~50 nm) due to the proceeding crystallization process. The room temperature spectrum of sample **7** can be fitted by two six-line hyperfine patterns with the nearly the same isomer shift (0.29 and 0.37 mm/s) and hyperfine magnetic fields of 48.6 and 51.4. Generally the isomer shift parameters are closed to those reported for CoFe_2_O_4_[77] and γ-Fe_2_O_3_ (0.27 mm/s) [78] or hematite, α-Fe_2_O_3_ (0.38 mm/s) [78]. Two sextets differing in isomer shift and hyperfine magnetic field can be ascribed to Fe(III) ions located at the tetrahedral (*T*_d_), δ = 0.37 mm/s, *H*_hyp_ = 51.4 T, and octahedral (*O*_h_), δ = 0.29 mm/s, *H*_hyp_ = 48.6 T, sites of the partially inverse spinel structure of CoFe_2_O_4_. In comparison with bulk CoFe_2_O_4_, (51.2 T for *T*_d_-site, 55.0 T for *O*_h_-site) the values of H_hyp_ of prepared CoFe_2_O_4_ nanoparticles (42.6 T for sample **6**, 48.6 and 51.4 T for sample **7**) are smaller due to their lower crystallinity [79].

**Figure 9:**
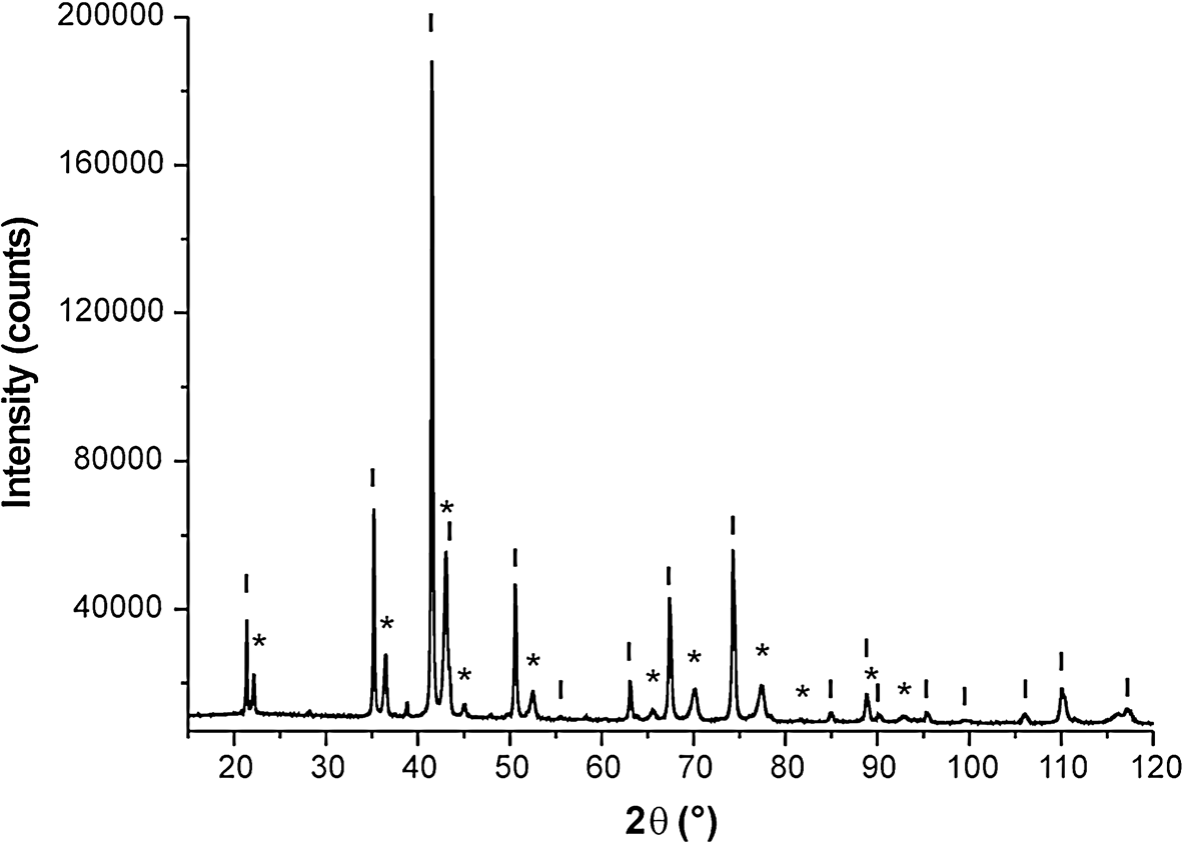
**XRD pattern of the sample prepared by heating of 1a up to 600°C.** (*) CoFe_2_O_4_, (|) Co_3_O_4_.

In Figure 9, the XRD spectrum of sample **7** is shown, which consists of two sets of peaks corresponding to two spinel phases. The presence of *γ*-Fe_2_O_3_ or α-Fe_2_O_3_ is clearly excluded because the first set of peaks corresponds to CoFe_2_O_4_ phase [77] (marked with asterisks) and the second pattern belongs to Co_3_O_4_ (labeled with short vertical lines). According to the XRD data, the molar ratio between these two phases was found to be 2.44 : 1 (CoFe_2_O_4_ : Co_3_O_4_) what is in accordance with the proposed stoichiometry of both phases.

**Figure 10:**
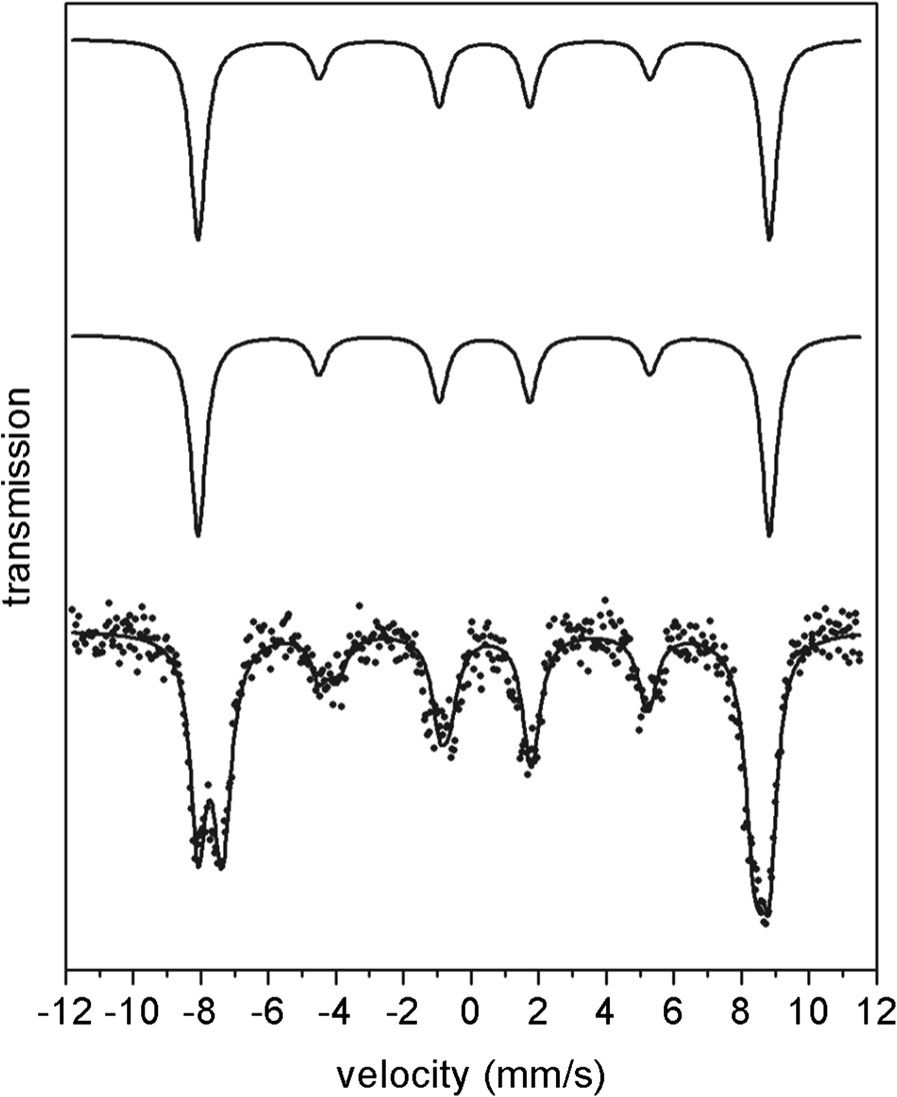
**In-field (5 K / 5 T) Mössbauer spectrum of the CoFe**_**2**_**O**_**4 **_**spinel phase formed by heating of 1a up to 600°C.** Crosses represent the experimental data and the upper and middle full lines represent the subspectra obtained from the fitting procedure for iron atoms in T- and O-sites, respectively, and the lower full line represents their sum. External field was applied parallel to the γ ray propagation.

To confirm the inverse spinel phase formed during the heating of **1a** up to 600°C and reveal the magnetic structure, the in-field Mössbauer spectrum of sample **7** was recorded (Figure 10, Table 5). It consists of two overlapping six-line hyperfine patterns corresponding to Fe(III) in *T*_d_- and *O*_h_-sites of spinel lattice (partial separation of both patterns). The data were successfully fitted with two sextets having the δ = 0.38 mm/s, ΔE_Q_ = −0.02 mm/s and *H*_hyp_ = 52.6 T corresponding to Fe(III) in *T*_d_-site and δ = 0.54 mm/s, ΔE_Q_ = −0.08 mm/s and *H*_hyp_ = 49.0 T for Fe(III) in *O*_h_-sites, respectively. The degree of conversion for inverse spinel, *x*, which identifies its formula Fe(III)_x_Co(II)_1–x_[Co(II)_x_Fe(III)_2–x_]O_4_ (brackets indicate ions at *O*_h_-site), can be calculated on the basis of the relative contribution of individual *T*_d_- and *O*_h_-site sextets. The value of *x* was obtained from the equation *x* = 2 *k*(1 + *k*) where *k* is the ratio between Fe(III) ions in the tetrahedral and octahedral sublattices calculated from the ratio between the areas of individual sextets corresponding to *T*_d_- and *O*_h_-sites. The obtained value of *x* = 0.9 indicates the formation of partially/almost fully inverse spinel structure Fe(III)_0.9_Co(II)_0.1_[Co(II)_0.9_Fe(III)_1.1_]O_4_. The determination of the degree of inversion at low temperature in external magnetic field is more accurate in comparison with room temperature spectra (Figure 8) because the applied magnetic field better separates the *T*_d_- and *O*_h_-site contributions (sextets) and the low temperature reduces the vibrations of the lattice and/or the Mössbauer nucleus (significantly influence the ration between the *T*_d_- and *O*_h_-signals).

**Table 5:** Hyperfine parameters of in-field Mössbauer spectrum of sample 7 at 5 K and in external field 5 T

**Position of the Fe**^**+3 **^**ions**	***δ*** **± 0.01 (mm/s)**	**Δ*E***_**Q**_ **± 0.01 (mm/s)**	***B***_**eff**_ **± 0.3 (T)**	**Γ ± 0.01 (mm/s)**	**RA ± 3%**	**A**_**2,5**_**/A**_**3,4**_	**Canting angle (°)**	**Average canting angle of both sites (°)**
T-site	0.38	−0.02	52.6	0.54	45	0.579	30.2	30.3
O-site	0.54	−0.08	49.0	0.63	55	0.589	30.4	

Where *δ* is center shift, Δ*E*_Q_ is quadrupole splitting, *B*_eff_ is effective magnetic field, *Γ* is line width and RA is the relative area of individual sextets. Furthermore, A_2,5_ and A_3,4_ are the intensities of the second/fifth, and third/fourth lines, respectively.

The intensities of 2nd and 5th lines are lower than expected for a thin sample with randomly oriented magnetic moments in the absence of magnetic field (ideally 3:2:1:1:2:3) which is characteristic for the materials in ferromagnetic and/or ferrimagnetic state and which corresponds to the partial alignment of the iron magnetic moments in the direction of the external magnetic field. If the iron magnetic moment were completely aligned, the 2nd and 5th lines would be completely vanished. Thus, the presence of the 2nd and 5th lines in the spectrum points out to the canting of Fe(III) spin in CoFe_2_O_4_ particles with respect to the direction of the external magnetic field. Based on the comparison of the areas corresponding to 2,5 and 1,6 lines, the average spin-canting angle, *θ*, was evaluated on the basis of the formula *θ* = arccos{[(4–s)/(4 + s)]^1/2^} [80] where s = A_2,5_/A_3,4_ and was calculated to be 30.3° (from both *T*_d_- and *O*_h_-sites). The thickness of the spin-canted surface layer of the sample was not deduced because the shape of prepared particles was not considered as spherical.

**Figure 11:**
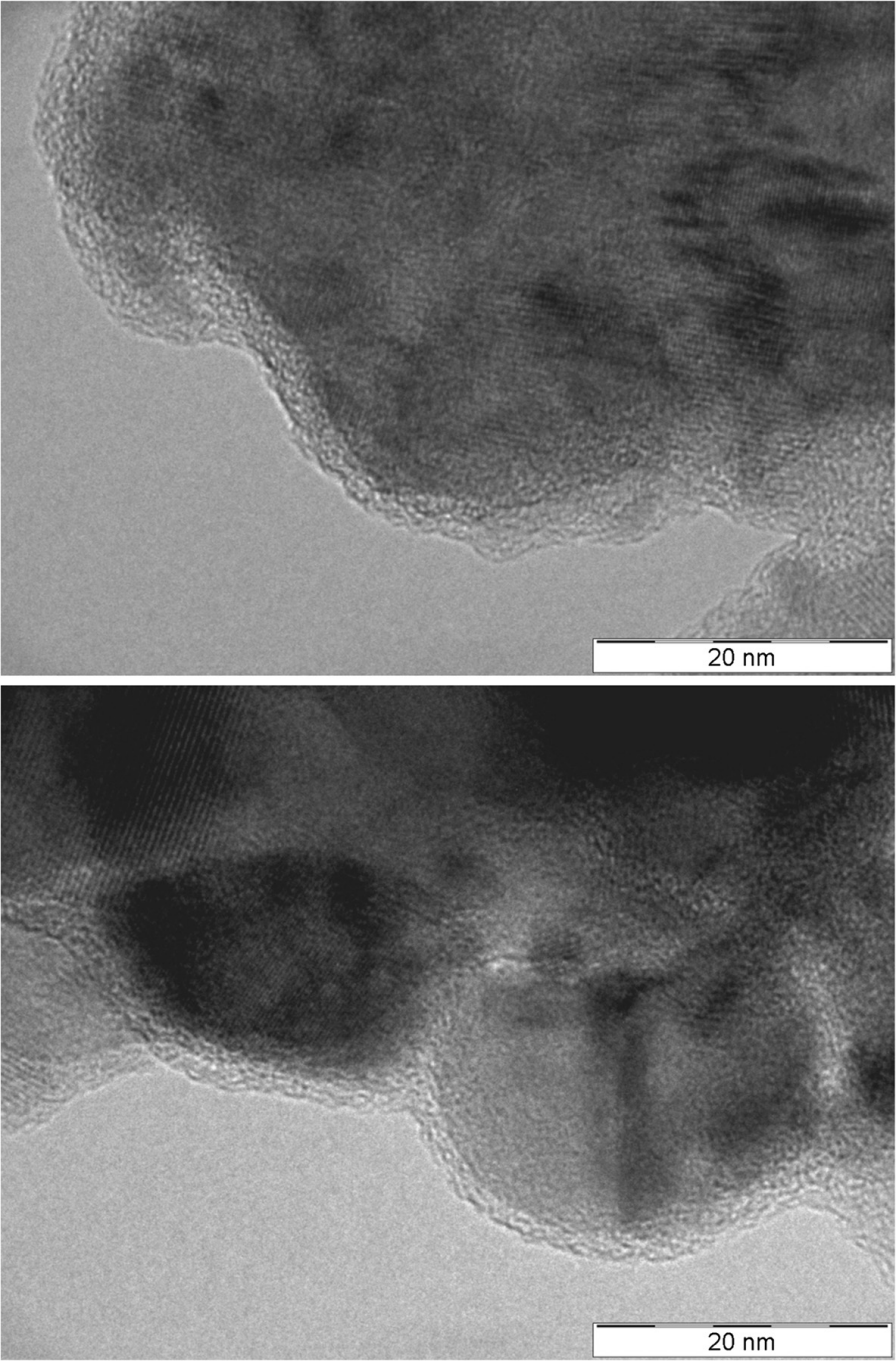
**TEM micrograph of the sample prepared by heating of 1a up to 600°C demonstrating the presence of CoFe**_**2**_**O**_**4**_**-Co**_**3**_**O**_**4 **_**in the form of nanocomposite particles.**

The morphology of the product formed by heating of **1a** up to 600°C (sample 7) is clearly visible on TEM pictures (Figure 11), which confirm the nanocomposite structure of the CoFe_2_O_4_–Co_3_O_4_ particles. But these nanoparticles, taken directly after the thermal decomposition, form agglomerates and are not well-dispersed and thus, their median size based on TEM analysis was not calculated.

## Conclusions

The thermal decomposition of [Co(*en*)_3_][Fe(CN)_6_]∙ 2H_2_O (**1a**) in air atmosphere was monitored by TG/DSC techniques. Seven samples were prepared by annealing **1a** at selected temperatures, which were completely characterized by elemental analyses, IR and ^57^Fe Mössbauer spectroscopies with the aim to elucidate the decomposition mechanism. It has been found that the topotactic dehydration of **1a** led to the formation of the hemihydrate [Co(*en*)_3_][Fe(CN)_6_]∙ 1/2H_2_O (**1b**), which was consequently decomposed in next four steps, going through the [Fe^II^(*en*)_2_(μ-NC)Co^III^(CN)_4_], [Fe^III^(NH_2_CH_2_CH_3_)_2_(μ-NC)_2_Co^II^(CN)_3_] and Fe^III^[Co^II^(CN)_5_] intermediates, leading to CoFe_2_O_4_-Co_3_O_4_ nanocomposite particles at as the final product (confirmed by TEM). It has been shown that a relatively small change regarding the heating gradient (5°C/min in this work *versus* 10°C/min in Ref. 52) may substantially influence the composition and particle size of the final product. Moreover, from a general point of view, the obtained results also revealed that the thermal decomposition of such a simple salt as [Co(*en*)_3_][Fe(CN)_6_]∙ 2H_2_O involves a relatively complicated mechanism driven mainly by the reaction conditions (such as the heating gradient and air atmosphere) and is composed by several overlapping steps and resulting in the formation of interesting nanoparticles with a nanocomposite structure of two magnetically different phases. It may be anticipated that the modulation of their properties would be possible by the exact control of the temperature (gradient or prolonged heating or amine-ligand substitution) and further investigation of magnetic properties of such system would be very interesting and helpful.

## Experimental

### Materials and reagents

K_3_[Fe(CN)_6_] (Merck) and *N,N’*-dimethylformamide (Lachema Brno) were purchased from commercial sources and used as received. [Co(*en*)_3_]Cl_3_∙ 3H_2_O was prepared by a slightly modified procedure described in the literature [81].

### Synthesis

[Co(*en*)_3_][Fe(CN)_6_]∙ 2H_2_O (**1a**).

0.39 g (1 mmol) of [Co(*en*)_3_]Cl_3_∙ 3H_2_O and 0.32 g (1 mmol) K_3_[Fe(CN)_6_] were added into 20 ml of *N,N’*-dimethylformamide (DMF) with stirring and refluxed for 1 h. The yellow-orange precipitate formed in the reaction mixture and the color of the solvent changed into light-green. The solid was filtered off, and consequently dissolved in 30 ml of warm water (60°C). The solution was left to stand at room temperature. Yellow-orange crystals were obtained by slow evaporation of the solvent after several hours. They were filtered off, washed with small amount of cold water and dried on air (yield 55%). *Anal. calc*. for C_12_H_28_N_12_CoFeO_2_: C, 29.8; H, 5.8; N, 34.7%. Found: C, 30.2; H, 5.5; N, 34.4%. IR: 3381 bm, 3279 s, 3232 bs, 3095 bs, 2117 vs, 2107 vs, 1630 m, 1609 m, 1578 m, 1464 m, 1399w, 1334 m, 1289 w, 1156 m, 1143 m, 1051 m, 763 w, 580 m.

### Physical measurements

Elemental analyses (C, H, N) were performed on a Flash EA 1112 Elemental Analyser (ThermoFinnigan). Thermogravimetric (TG) and differential scanning calorimetry (DSC) measurements of **1a** were performed in static air atmosphere under dynamic heating conditions with a sample weight of 6–8 mg and heating rate 5°C/min. DSC measurements were performed in the range of 20–400°C on a DSC XP-10 analyser (THASS, GmbH) and TG curve was measured between 20–500°C on a TG XP-10 instrument (THASS, GmbH). To monitor the intermediates of the thermal decay, the samples were prepared in conditions of DSC measurement by dynamic heating up to the selected temperatures, with the heating rate of 5°C/min and consequently analyzed by suitable techniques. The transmission ^57^Fe Mössbauer spectra were collected at 300 K in a constant acceleration mode with a ^57^Co(Rh) source. The final decomposition product was also analyzed at 5 K in zero external magnetic field and at 5 K in an external magnetic field of 5 T, applied parallel to the X-ray direction using a cryomagnetic system of Oxford Instruments. The isomer shift values were calibrated against metallic α-Fe. In-field Mössbauer spectra were fitted by means of the Lorentzian line shapes using the least squares method featured in the MossWinn computer program. FT IR spectra were measured on a Nexus 670 FT-IR (Thermo Nicolet) spectrophotometer using KBr pellets in the wavenumber range of 400–4000 cm^–1^ at temperatures 25, 150, 230, 265 and 450°C.

The transmission electron microscopy was performed on a JEOL JEM-2010 instrument (LaB_6_ cathode; accelerating voltage of 200 kV; point-to-point resolution 0.19 nm) to consider the particle size and structure of the final spinel phase. A drop of high-purity distilled water, containing the ultrasonically dispersed particles, was placed onto a holey-carbon film supported by a copper-mesh TEM grid and dried in air at room temperature.

### X-ray structure determinations and refinements

X-ray single crystal diffraction experiments for both **1a** and **1b** were carried out on a four circle κ-axis Xcalibur2 diffractometer at 100(2) K equipped with a CCD detector Sapphire2 (Oxford Diffraction). The CrysAlis software package [82] was used for data collection and reduction. The structures were solved by the SIR97 program [83] incorporated within the WinGX program package [84]. All non-hydrogen atoms of **1a** and **1b** were refined anisotropically by the full-matrix least-squares procedure [85], with weights: w = 1/[σ^2^(F_o_^2^) + (0.0633P)^2^] for (**1a**) and w = 1/[σ^2^(F_o_^2^) + (0.0605P)^2^ + 0.3587P] for (**1b**), where P = (F_o_^2^ + 2F_c_^2^)/3. Hydrogen atoms were found in the difference Fourier maps, nevertheless these were modeled using a riding model, except for those belonging to crystal water molecules, which parameters were fixed. The structures of **1a** and **1b**, depicted in Figures 1, 2, 6 and 7, were drawn using the Diamond software [86]. X-ray powder diffraction pattern of the final decomposition product was recorded using a PANalytical X’Pert MPD device (iron-filtered Co*K*_α_ radiation: λ = 0.178901 nm, 40 kV and 30 mA) in Bragg-Brentano geometry, and equipped with X’Celerator detector. Powdered sample was spread on zero-background single-crystal Si slides and scanned in continuous mode (resolution of 0.017° 2 Theta, scan speed of 0.005° 2 Theta per second) within the angular range 10-120^o^. The acquired patterns were processed (i.e., phase analysis and Rietveld refinement) using X´ Pert HighScore Plus software (PANalytical, The Netherlands), in combination with PDF-4+ and ICSD databases (ICSD collection codes are: Co_3_O_4_ – 63165, CoFe_2_O_4_ – 98553).

## Abbreviations

aed: *N*,*N*’-bis(3-aminopropyl)-ethylenediamine; bipy: 2-(pyridin-2-yl)pyridine = 2,2^′^-bipyridine; bipy2: 4-(pyridin-4-yl)pyridine = 4,4^′^-bipyridine; DSC: Differential scanning calorimetry; EGA: Evolved gas analysis; en: Ethylenediamine = ethane-1,2-diamine; DMF: *N,N’*-dimethylformamide; IR: Infrared; ox: Oxalate; PBA: Prussian blue analogue; TEM: Transmission electron microscopy; terpy: 2,6-di(pyridin-2-yl)pyridine = 2,2^′^:6^′^,2^′^-terpyridine; TG: Thermogravimetry; tren: Tris(2-aminoethyl)amine; XRD: X-ray powder diffraction.

## Competing interests

The authors declare that they have no competing interests.

## Authors’ contributions

MMM synthesized all samples and made substantial contributions to the data collection and analysis. BD made substantial contributions to the survey results, data analysis/interpretation and drafting the manuscript. ZT performed single-crystal diffraction experiments and calculations, and together with RZ participated in the overall data interpretations and were involved in drafting the manuscript. BD, ZT, RZ and JČ revised the manuscript for the intellectual content. All authors read and approved the final version of the manuscript.

## Supplementary Material

Additional file 1 **Electronic Supplementary Information.** The file contains IR spectrum of 1a (**Figure S1**) and temperature dependences of the effective magnetic moment (*μ*_eff_) and reciprocal molar susceptibility (1/*χ*_mol_) (Additional file 1: Figures S2 and S3). Click here for file

Additional file 2 A CIF file for complex 1a. Click here for file

Additional file 3 A CIF file for complex 1b. Click here for file
