# Supplementary material for: Thermal decomposition of [Co(en)3][Fe(CN)6]∙ 2H2O: Topotactic dehydration process, valence and spin exchange mechanism elucidation
Source: Chem Cent J. 2013 Feb 8;7:28. doi: 10.1186/1752-153X-7-28 (PMC3637566; doi:10.1186/1752-153X-7-28)
Supplement: Additional file 1 — Electronic Supplementary Information. The file contains IR spectrum of 1a (Figure S1) and temperature dependences of the effective magnetic moment (μeff) and reciprocal molar susceptibility (1/χmol) (Additional file 1: Figures S2 and S3). [file 1752-153X-7-28-S1.doc]

**Electronic Supplementary Information**

for

**Thermal decomposition of [Co(*en*)_3_][Fe(CN)_6_]∙2H_2_O: Topotactic dehydration process, valence and spin exchange mechanisms elucidation**

Zdeněk Trávníček^1§^, Radek Zbořil^2^, Miroslava Matiková-Maľarová^1^, Bohuslav Drahoš^1^, Juraj Černák^3^

^1^ Regional Centre of Advanced Technologies and Materials & Department of Inorganic Chemistry, Faculty of Science, Palacký University, Tř. 17. listopadu 12, CZ-77146 Olomouc, Czech Republic

^2^ Regional Centre of Advanced Technologies and Materials, Department of Physical Chemistry, Faculty of Science, Palacký University, Tř. 17. listopadu 12, CZ-77146 Olomouc, Czech Republic

^3^ Department of Inorganic Chemistry, Institute of Chemistry, Faculty of Science, P.J. Šafárik University in Košice, Moyzesova 11, SK-041 54 Košice, Slovakia

**Content:**

p. 1 **Figure S1** IR spectrum (4000–400 cm^–1^) of **1a** measured at room temperature.

p. 2 **Figure S2** Temperature dependence of the effective magnetic moment (*μ*_eff_) of **1a** and **1b**.

p. 3 **Figure S3** Temperature dependence of the reciprocal molar susceptibility of **1a** and **1b** fitted by the Curie-Weiss law in the temperature range of 50–300 K.


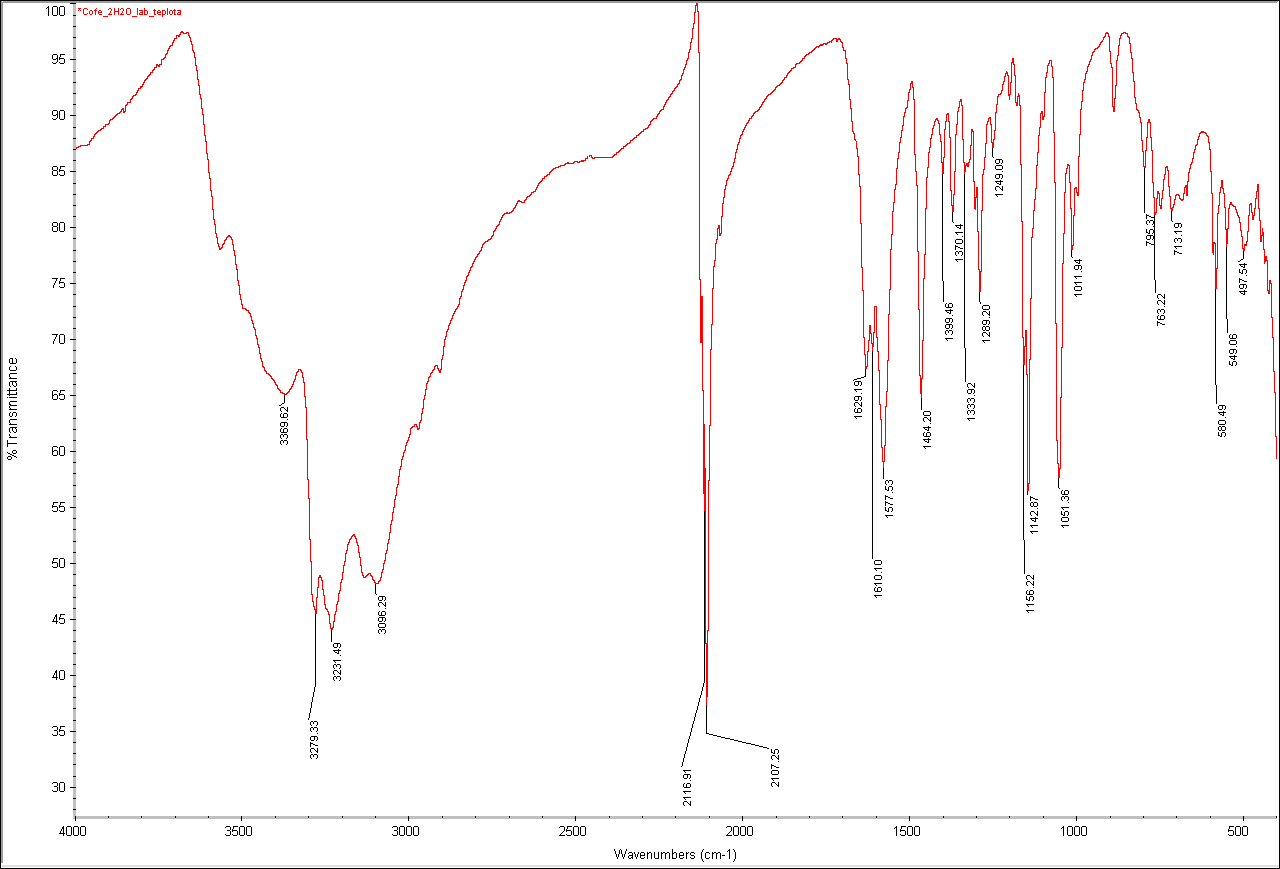


**Figure S1** IR spectrum (4000–400 cm^–1^) of **1a** measured at room temperature.


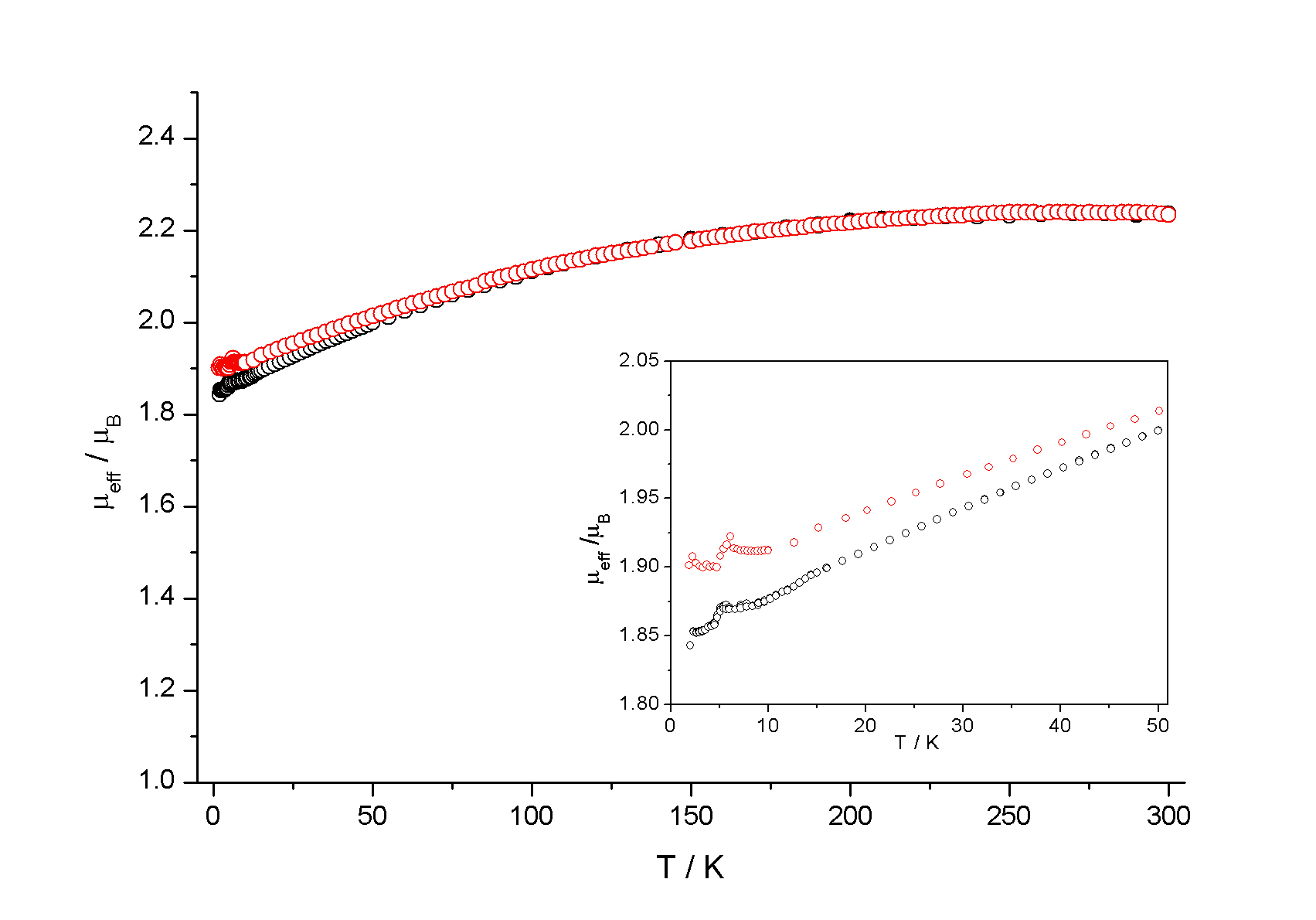


**Fig. S2** Temperature dependence (5–300 K) of the effective magnetic moment of **1a** (black) and **1b** (red). The inset is zoom of 5–50 K region and is given for better comparison of the difference between **1a** (black) and **1b** (red) at lower temperature.

**Fig. S3** Temperature dependence of the reciprocal molar susceptibility of **1a** (up) and **1b** (down) fitted by the Curie-Weiss law in the temperature range from 50 to 300 K. Empty circles – experimental data, full line – calculated data with *g* = 2.67, Θ = -19 K for *S* = 1/2 for **1a** and *g* = 2.77, Θ = -18 K for *S* = 1/2 for **1b**.
